# Distinct virologic trajectories in chronic hepatitis B identify heterogeneity in response to nucleos(t)ide analogue therapy

**DOI:** 10.1016/j.jhepr.2024.101229

**Published:** 2024-10-09

**Authors:** Tingyan Wang, Cori Campbell, Alexander J. Stockdale, Stacy Todd, Karl McIntyre, Andrew Frankland, Jakub Jaworski, Ben Glampson, Dimitri Papadimitriou, Luca Mercuri, Erik Mayer, Christopher R. Jones, Hizni Salih, Gail Roadknight, Stephanie Little, Theresa Noble, Kinga A. Várnai, Cai Davis, Ashley I. Heinson, Michael George, Florina Borca, Louise English, Luis Romão, David Ramlakhan, NIHR HIC Viral Hepatitis and Liver Disease Consortium, Eleanor Barnes, Philippa C. Matthews, William Gelson, Graham S. Cooke, Salim I. Khakoo, Eleni Nastouli, Jim Davies, Kerrie Woods, Alexander J. Stockdale, Stephen Ryder, Ahmed Elsharkawy, Nicholas Easom, William Bernal, Shazaad Ahmad, Douglas Macdonald, Tingyan Wang, Cori Campbell, Simon Stanworth, Suzanne Maynard, Gail Roadknight, Stephanie Little, Kinga A. Várnai, Ben Glampson, Dimitri Papadimitriou, Luca Mercuri, Christopher R. Jones, Jakub Jaworski, Cai Davis, Florina Borca, Ashley Heinson, Michael George, Heidi MacNaughton, Yun Kim, Josune Olza Meneses, Louise English, Timothy Roberts, Luis Romão, David Ramlakhan, Stacy Todd, Heather Rogers, Karl McIntyre, Andrew Frankland, Hizni Salih, Theresa Noble, Lara Roberts, Finola Higgins, Javier Vilar, Ruth Norris, George Tilston, Ilina Serafimova, Sarah Montague, Juliette Verheyden, Irene Juurlink, Kathryn Jack, Alex Waldren-Glenn, Lizzie Poole, Victoria Day, Berit Reglar, Kerrie Woods, Jim Davies, Eleni Nastouli, Salim I. Khakoo, William Gelson, Graham S. Cooke, Eleanor Barnes, Philippa C. Matthews

**Affiliations:** 1 NIHR Oxford Biomedical Research Centre, Oxford, UK; 2 Nuffield Department of Medicine, University of Oxford, Oxford, UK; 3 Department of Clinical Infection, Microbiology and Immunology, Institute of Infection, Veterinary and Ecological Sciences, University of Liverpool, Liverpool, UK; 4 Tropical Infectious Diseases Unit, Royal Liverpool Hospital, Liverpool University Hospitals NHS Trust, Liverpool, UK; 5 Liverpool Clinical Laboratories, Liverpool University Hospitals NHS Trust, Liverpool, UK; 6 Cambridge University Hospitals NHS Foundation Trust, Cambridge, UK; 7 iCARE Secure Data Environment, Digital Collaboration Space, Imperial College Healthcare NHS Trust, London, UK; 8 Department of Surgery and Cancer, Imperial College London, London, UK; 9 Department of Infectious Disease, Imperial College London, London, UK; 10 NIHR Health Informatics Collaborative, Oxford University Hospitals NHS Foundation Trust, Oxford, UK; 11 Southampton Emerging Therapies and Technologies Centre, University Hospital Southampton NHS Foundation Trust, Southampton, UK; 12 Clinical Informatics Research Unit, Faculty of Medicine, University of Southampton, Southampton, UK; 13 NIHR University College London Hospitals Biomedical Research Centre, London, UK; 14 Department of Computer Science, University of Oxford, Oxford, UK; 15 Department of Infection, Immunity and Inflammation, UCL Great Ormond Street Institute of Child Health, London, UK; 16 Department of Virology, UCLH, London, UK; 17 School of Clinical and Experimental Sciences, Faculty of Medicine, University of Southampton, Southampton, UK; 18 Cambridge Liver Unit, Cambridge University Hospitals NHS Foundation Trust, Cambridge, UK; 19 Faculty of Medicine, Department of Infectious Disease, Imperial College London, UK; 20 The Francis Crick Institute, London, UK; 21 Division of Infection and Immunity, University College London, London, UK; 22 Department of Infectious Diseases, University College London Hospital, London, UK

**Keywords:** HBV, Viral load, Longitudinal, Liver fibrosis, Cirrhosis, Antiviral treatment, Nucleotide analogue, Latent class mixed models, Health Informatics Collaborative (HIC)

## Abstract

**Background & Aims:**

The dynamics of HBV viral load (VL) in patients with chronic hepatitis B (CHB) on nucleos(t)ide analogue (NA) treatment and its relationship with liver disease are poorly understood. We aimed to study longitudinal VL patterns and their associations with CHB clinical outcomes.

**Methods:**

Utilising large scale, routinely collected electronic health records from six centres in England, collated by the National Institute for Health and Care Research Health Informatics Collaborative (NIHR HIC), we applied latent class mixed models to investigate VL trajectory patterns in adults receiving NA treatment. We assessed associations of VL trajectory with alanine transaminase, and with liver fibrosis/cirrhosis.

**Results:**

We retrieved data from 1,885 adults on NA treatment (median follow-up 6.2 years, IQR 3.7–9.3 years), with 21,691 VL measurements (median 10 per patient, IQR 5–17). Five VL classes were identified from the derivation cohort (n = 1,367, discrimination: 0.93, entropy: 0.90): class 1 ‘long term suppression’ (n = 827, 60.5%), class 2 ‘timely virological suppression’ (n = 254, 18.6%), class 3 ‘persistent moderate viraemia’ (n = 140, 10.2%), class 4 ‘persistent high-level viraemia’ (n = 44, 3.2%), and class 5 ‘slow virological suppression’ (n = 102, 7.5%). The model demonstrated a discrimination of 0.93 and entropy of 0.88 for the validation cohort (n = 518). Alanine transaminase decreased variably over time in VL-suppressed groups (classes 1, 2, 5; all *p* <0.001), but did not significantly improve in those with persistent viraemia (classes 3, 4). Patients in class 5 had twofold increased hazards of fibrosis/cirrhosis compared with class 1 (adjusted hazard ratio, 2.00; 95% CI, 1.33–3.02).

**Conclusions:**

Heterogeneity exists in virological response to NA therapy in CHB patients, with over 20% showing potentially suboptimal responses. Slow virological suppression is associated with liver disease progression.

**Impact and implications:**

Treatment recommendations for people living with chronic hepatitis B virus (HBV) infection are becoming less stringent, meaning that more of the population will be eligible to receive therapy with nucleos(t)ide analogue agents. We explored outcomes of HBV treatment in a large UK dataset, describing different responses to treatment, and showing that the viral load is not completely suppressed after 1 year in about one in five cases, associated with an increased risk of liver complications. As treatment is rolled out more widely, patients and clinicians need to be aware of the potential for incomplete virologic responses. The findings can support the identification of high-risk individuals, improve early fibrosis and cirrhosis prediction, guide monitoring and preventive interventions, and support public health elimination goals.

## Highlights

• Virologic responses to NA therapy in patients with chronic hepatitis B show significant variability.
• Five distinct virologic trajectory classes with distinct clinical, laboratory and demographic features are identified.
• Over 20% of patients with chronic hepatitis B exhibit suboptimal virological responses to NA treatment.
• Complete virological suppression for patients with chronic hepatitis B on current standard antiviral treatment can be slow.
• Slow virological suppression is associated with a twofold increased risk of fibrosis/cirrhosis.

## Graphical abstract

**Figure:**
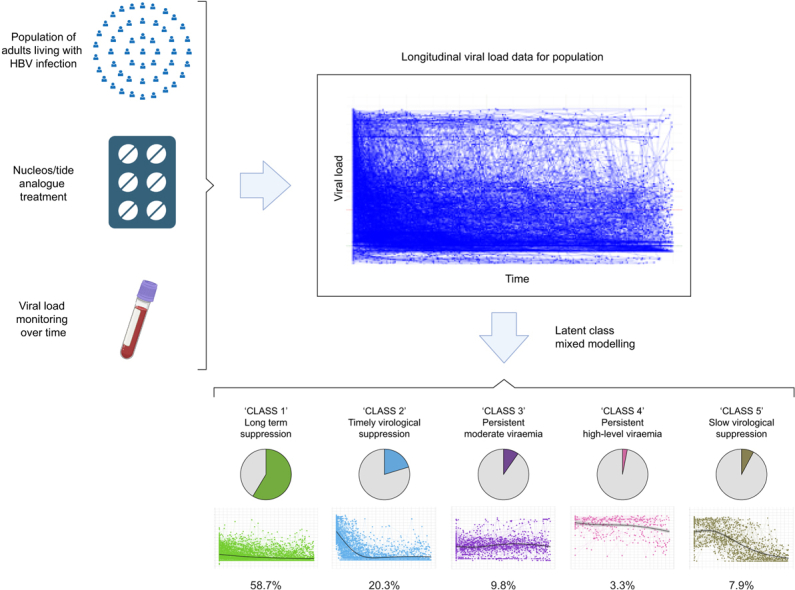

## Introduction

Chronic hepatitis B (CHB) is a significant international public health concern, affecting nearly 300 million individuals worldwide, and accounting for a large proportion of the global burden of cirrhosis and hepatocellular carcinoma (HCC).^1^ In 2019, the World Health Organization (WHO) estimated there were approximately 1.5 million new infections and 820,000 deaths related to hepatitis B virus (HBV) worldwide,^2^^,^^3^ while a new global report released in 2024 documents 254 million people worldwide living with CHB, and headlines increasing mortality since 2019.^4^ International sustainable development goals for the elimination of HBV as a public health threat by 2030 aim for 65% reduction in mortality and 90% reduction in incidence compared with baseline levels observed in 2015.^5^ Successful deployment of oral antiviral treatment is a cornerstone of interventions required to achieve these ambitious targets.

Long-term antiviral treatment with nucleos(t)ide analogue (NA) agents suppresses HBV replication and reduces the long-term risk of inflammatory liver disease, cirrhosis, and HCC.^6^^,^^7^ However, complete virologic suppression can be slow,[8], [9], [10], [11][8], [9], [10], [11] potentially taking up to 2–3 years for HBV DNA viral load (VL) to suppress below the limit of detection in blood. Furthermore, in a minority of individuals, there is a risk of persistent viraemia or viral rebound,[8], [9], [10], [11], [12], [13][8], [9], [10], [11], [12], [13] which might be associated with high pre-treatment HBV DNA levels,^8^ HBeAg positivity at baseline,^9^^,^^10^ HIV coinfection,^12^ incomplete treatment adherence,^13^ and/or HBV resistance to antiviral therapy.^14^^,^^15^ Although studies have investigated the overall pattern of changes in VL (e.g. time to virological response, the proportion of non-suppressed) during antiviral treatment,[8], [9], [10], [11][8], [9], [10], [11] the detailed characterisation of longitudinal VL trajectory patterns has been limited, and largely based on clinical trial cohorts which may not represent real-world populations.

At present, only a minority of the population with CHB are deemed eligible for NA treatment, based on guidelines for HBV management which use clinical and laboratory assessments to identify those at highest risk.^6^^,^^7^^,^^16^^,^^17^ However, such guidance is changing over time, recognising the potential benefits of relaxed and simplified treatment criteria, as exemplified by new recommendations in China which suggest treatment for all those with detectable HBV DNA and alanine transaminase (ALT) above the upper limit of normal (ULN)^18^ and guidelines from the WHO released in March 2024 which also move to more inclusive treatment.^19^ As an increasing number of individuals embark on NA therapy, there is a pressing need to understand therapeutic responses, and to identify and interpret the clinical significance of situations in which virologic suppression is inadequate.

There are recommendations in European^17^ and UK guidelines^7^ for supporting management of patients in whom viraemia is persistent or rebounds on NA treatment. These suggest reviewing and supporting adherence in people taking tenofovir disoproxil fumarate (TDF) in whom virological suppression is not achieved (particularly in the setting of advanced liver disease) or in whom there is not an ongoing downward trajectory of VL over time. In such instances, adding lamivudine (LAM) or entecavir (ETV) to tenofovir can also be considered.^17^ However, there is a lack of evidence or structured algorithms to determine the optimal timing for assessing the impact of such an intervention (e.g. 48 *vs.* 96 weeks) and to establish a consistent view over an acceptable VL threshold to be attained on treatment (e.g. HBV DNA <2,000 IU/ml). Thus, more evidence is needed to identify patients at risk of inadequate virologic suppression, to inform investigations or interventions, and to assess the public health consequences of incomplete treatment response.

Latent class mixed modelling, a contemporary unsupervised approach, has already demonstrated clinical relevance in various disease areas, including cardiovascular disease,^20^ chronic kidney disease,^21^ human immunodeficiency virus (HIV),^22^ and coronavirus disease (caused by SARS-CoV-2 infection),^23^ by disaggregating sub-phenotypes. We used this approach to avoid any prior assumptions about HBV VL trajectories and to take an unbiased approach to identify distinct on-treatment groups. We set out to take advantage of repeated HBV VL measurements to describe the VL trajectory over time and explore its prognostic value in a longitudinal dataset, using large-scale clinical data collected from secondary care services in the UK through the National Institute for Health and Care Research (NIHR) Health Informatics Collaborative (HIC) programme.

Our objectives include (a) characterising HBV virologic trajectory patterns in adults receiving NA therapy using electronic patient records (EPRs), (b) stratifying demographic, laboratory, and clinical characteristics by VL trajectory pattern, (c) assessing liver inflammation across VL trajectory patterns, and (d) determining whether identified VL patterns were associated with liver disease progression. We aimed to provide insights into the dynamics of HBV virologic trajectories during NA therapy, which are relevant to supporting the management of individuals on treatment, facilitating the development of personalised treatment approaches, and providing evidence that can inform public health approaches.

## Patients and methods

### Data collection

We used routinely collected clinical data from 10,460 individuals under longitudinal follow up for HBV infection in secondary care services. These were collated from six National Health Service (NHS) trusts (distinct regional organisations, each a separate legal entity, responsible for provision and commissioning of health care, and each made up of several hospitals) in England, established through the NIHR HIC Viral Hepatitis and Liver Disease theme: Cambridge University Hospitals NHS Foundation Trust (CUH), Imperial College Healthcare NHS Trust (ICHT), Liverpool University Hospitals NHS Foundation Trust (LUH), Oxford University Hospitals NHS Foundation Trust (OUH), University College London Hospitals NHS Foundation Trust (UCLH), and University Hospital Southampton NHS Foundation Trust (UHS). Data were collated by a local NIHR HIC team in each site, being drawn from operational systems including EPR systems into a data warehouse and linked to produce a comprehensive record for each patient with a data validation process, as described in a previous methods paper and cohort profile.^24^^,^^25^ Data were shared with the host organisation (OUH), validated against the defined data model and then integrated into the central database for research studies. The management of the central database is governed by the NIHR HIC Data Sharing Framework.

The data elements used for this study included demographics, deprivation scores (Index of Multiple Deprivation [IMD]^26^), laboratory tests (liver biochemistry, other tests reflecting liver health, identification of other chronic viral infections) (Table S1), HBV treatment regimen information, imaging, liver biopsy reports, and ICD-10 diagnosis codes for inferring liver fibrosis and cirrhosis status.

### Eligibility criteria

To be included in this study, all of the following criteria had to be met: (a) HBV infection (positive HBsAg or detectable HBV DNA test), (b) any record of antiviral treatment with NA (monotherapy or combination) therapy, including TDF or tenofovir alafenamide (TAF), ETV, and older regimens, e.g. LAM, adefovir (ADV), (c) two or more quantified HBV DNA VL measurements spanning ≥6 months during the period of treatment, and (d) aged ≥18 years at the first quantified HBV DNA VL.

### Statistical analysis

We aimed to use 70% of our sample for model development, and 30% for validation, and thus based on the number of patients for each site, data from four sites (ICHT, LUH, UCLH, UHS) were collectively utilised as the derivation set ensuring a sufficient sample size for modelling, while data from the remaining two sites (CUH, OUH) were exclusively reserved for external validation. We defined the date of first VL measurement during the period of treatment as ‘baseline’, as earliest treatment start dates were not reliably recorded in the EPR systems. All analyses for this study were conducted in R 4.2.1 (R Foundation for Statistical Computing, Vienna, Austria). We conducted latent class mixed modelling^27^ to characterise VL trajectories using the *lcmm* package (version 2.0.2).

We applied a nonlinear (log) transformation to HBV DNA VL levels before modelling. Varied numbers of VL measurements at different time points were considered in the approach by specifying fixed-effects at the latent process level and class-specific level, as well as including random-effects for individual patients. We explored modelling with different functions of time (linear, quadratic, splines) and finally determined natural splines on time with internal knots (at first and second tertiles) and boundary knots (at 2.5th and 97.5th percentiles) as the most appropriate function of time. In the latent class mixed models, we also included age, sex, and ethnicity as covariates in the class-membership multinomial logistic model. Age was included by performing splines with an internal knot and boundary knots. For models with more than one class, we randomly generated the initial values from the asymptotic distribution of the estimates of the one-class model. We derived a set of models with a varying number of latent classes, and the optimal number of VL trajectory patterns (which we defined as ‘classes’) was determined by the Bayesian information criteria (BIC), the Akaike information criteria (AIC), the discrimination, the relative entropy, the odds of correct classification, and the interpretability of the model.^21^^,^^28^^,^^29^ The calculation of discrimination and entropy^30^ is provided in Table S2.

We assessed and interpreted the clinical meaningfulness of the VL trajectory classes by the following approaches: (a) tabulating patients’ characteristics by latent classes; (b) assessing the clinical plausibility of the trajectory classes by analysing alanine transaminase (ALT) trend for each; (c) investigating associations with outcomes.

We tabulated the demographic, laboratory, and treatment characteristics at baseline across the identified classes in derivation and validation cohorts. For categorical variables, Χ^2^ or Fisher’s exact tests were used for comparison. For continuous variables, *t* or Wilcoxon tests were used for two group comparisons, whilst ANOVA or the Kruskal-Wallis test for comparison of more than two groups. All significance tests performed were two-sided.

To evaluate the classification of VL trajectories using another biomarker, we used ALT, which reflects liver inflammation.^29^ To determine whether persistent viraemia drives liver inflammation, we combined data from derivation and validation cohorts, and performed linear mixed effects modelling with random intercepts and slopes to assess longitudinal trends of ALT levels for these distinct patient classes. All linear mixed effects models were adjusted for demographics including age, sex, and ethnicity. We also provided distribution and stratification of ALT levels at baseline, and at 12, 24, 36, 48, 60, and 72 months after the baseline, in the five classes.

As described previously,^25^ we identified liver fibrosis and cirrhosis status based on the following data sources: Ishak or METAVIR scores from biopsy reports, liver stiffness measurements from transient elastography (FibroScan, EchoSens, Paris, France) if available, imaging reports (ultrasound, CT, and MRI), ICD diagnosis information, or on aspartate aminotransferase (AST) to platelet ratio index (APRI) or Fibrosis-4 (FIB-4) scores (which were calculated by laboratory tests using measurements of platelets, AST, and ALT measured within 12 weeks of each other). We used pre-defined thresholds for significant/advanced fibrosis and cirrhosis: 1.5 and 2.0 for APRI score, respectively; 3.25 and 3.6 for FIB-4 score, respectively.^31^^,^^32^ We used APRI and FIB-4 as surrogate markers for determining fibrosis or cirrhosis only when biopsy, FibroScan, ultrasound/CT/MR, or ICD data were not available. If either APRI or FIB-4 suggested fibrosis/cirrhosis, a patient will be classified as fibrosis/cirrhosis. For the analysis of associations of VL trajectory classes with liver fibrosis and cirrhosis, we excluded individuals with no data available for liver fibrosis/cirrhosis diagnosis. To explore liver disease progression for patients with different VL trajectory classes, patients with fibrosis and cirrhosis occurring within 6-months of their first VL measurement (baseline) or follow up of liver disease status <6 months were excluded to avoid immortal time bias.

We compared the presence of liver fibrosis and/or cirrhosis in the overall cohorts (combining derivation and validation cohorts according to the VL trajectory classes). We performed Kaplan–Meier (K–M) analysis to compare the probability of being fibrosis and cirrhosis-free over time between classes. We used univariate and multivariate Cox proportional hazards models to investigate whether the distinct virologic classes predicted disease progression to liver fibrosis/cirrhosis, reporting 95% confidential intervals (CIs). To handle missingness on covariates, multiple imputations for missing data were performed using the MICE algorithm.^33^ We conducted Cox proportional-hazards modelling with the imputed datasets and pooled the estimates. We performed sensitivity analysis to investigate the robustness of associations observed. We performed subgroup analyses if applicable. We undertook receiver operating characteristic (ROC) analysis using the *pROC* package (version 1.18.5)^34^ to examine the predictive relationship between VL trajectory and liver fibrosis/cirrhosis, compared with that of other predictors of liver fibrosis and cirrhosis identified by multivariate analysis, reporting the area under the curve (AUC), sensitivity, specificity, and accuracy.

To explore the utility of the developed VL trajectory classification model for facilitating patient stratification in the real world, we conducted sensitivity analysis on the validation cohort with different scenarios, by changing the number of HBV DNA VL measurements per patient. We also assessed the performance of classification with and without restrictions on the minimum follow-up duration of HBV DNA VL.

## Results

### Characteristics of study cohort

We included 1,885 adults receiving NA treatment for chronic HBV infection, 62.8% male with median age 43 years (IQR 34–54). Longitudinal VL data were available over a median follow-up duration of 6.2 years (IQR 3.7–9.3 years). We analysed a total of 21,691 VL measurements, with a median of 10 measurements per patient (IQR 5–17, range 2–45). The flow chart of participant selection is illustrated in Fig. S1. Baseline characteristics and follow-up information of the derivation and validation cohorts are presented in Table 1. Missingness of some laboratory parameters varied by sites (e.g. AST and HBeAg), reflecting differences in laboratory testing practice.

**Table 1:** Follow-up duration, demographics, and medical characteristics at presentation of adults with chronic HBV infection on NA therapy in the overall study cohort, and the derivation and validation cohorts.

Parameter	Overall study cohort	Derivation cohort	Validation cohort
Number of patients	1,885	1,367	518
Follow-up duration, years	6.2 [3.7–9.3]	6.6 [3.8–9.4]	5.7 [3.5–7.4]
Total number of VL measurements	21,691	16,980	4,711
Number of VL measurements per patient	10 [5-17]	11 [6-18]	8 [4-12]
Sex, male	1,184 (62.8)	846 (61.9)	338 (65.3)
Age, years	43 [34, 54]	44 [34, 55]	41 [33, 52]
Age group, years			
18–24	77 (4.1)	55 (4.0)	22 (4.2)
25–34	442 (23.4)	304 (22.2)	138 (26.6)
35–44	498 (26.4)	338 (24.7)	160 (30.9)
45–54	409 (21.7)	315 (23.0)	94 (18.1)
55–64	284 (15.1)	206 (15.1)	78 (15.1)
65–74	131 (6.9)	113 (8.3)	18 (3.5)
≥75	44 (2.3)	36 (2.6)	8 (1.5)
Ethnic group			
Asian	624 (39.4)	418 (36.7)	206 (46.3)
Black	314 (19.8)	243 (21.4)	71 (16.0)
White	393 (24.8)	260 (22.8)	133 (29.9)
Mixed/other ethnicity	252 (15.9)	217 (19.1)	35 (7.9)
Not reported	302 (16.0)	229 (16.8)	73 (14.1)
IMD (decile)	5 [3, 7]	4 [2, 6]	6 [4, 9]
IMD category			
20% most deprived	415 (24.4)	367 (30.3)	48 (9.8)
20% to 40%	405 (23.8)	320 (26.4)	85 (17.3)
40% to 60%	375 (22.0)	261 (21.6)	114 (23.3)
60% to 80%	293 (17.2)	183 (15.1)	110 (22.4)
20% least deprived	213 (12.5)	80 (6.6)	133 (27.1)
Not reported	184 (9.8)	156 (11.4)	28 (5.4)
HBeAg status			
Negative	814 (66.3)	541 (67.1)	273 (64.7)
Positive	414 (33.7)	265 (32.9)	149 (35.3)
Not available	657 (34.9)	561 (41.0)	96 (18.5)
Anti-HBe status			
Negative	460 (35.5)	305 (34.5)	155 (37.5)
Positive	836 (64.5)	578 (65.5)	258 (62.5)
Not available	589 (31.2)	484 (35.4)	105 (20.3)
HBV VL, log_10_ IU/ml	3.0 [1.5, 5.3]	2.7 [1.5, 5.0]	3.6 [1.9, 5.8]
HBV VL category			
<20 IU/ml	166 (8.8)	120 (8.8)	46 (8.9)
20 to <2,000 IU/ml	842 (44.7)	653 (47.8)	189 (36.5)
2,000 to <20,000 IU/ml	231 (12.3)	155 (11.3)	76 (14.7)
≥20,000 IU/ml	646 (34.3)	439 (32.1)	207 (40.0)
ALT, IU/L	35 [22, 58]	34 [21, 57]	37 [25, 60]
ALT, not available	123 (6.5)	87 (6.4)	36 (6.9)
AST, IU/L	33 [25, 48]	32 [25, 46]	35 [26, 52]
AST, not available	848 (45.0)	475 (34.7)	373 (72.0)
Platelets, 10^9^/L	203 [159, 246]	201 [158, 246]	208 [164, 246]
Platelets, not available	247 (13.1)	211 (15.4)	36 (6.9)
Albumin, g/L	40 [37, 44]	41 [37, 44]	40 [37, 44]
Albumin, not available	236 (12.5)	201 (14.7)	35 (6.8)
ALP, IU/L	78 [62, 101]	76 [60, 96]	85 [65, 142]
ALP, not available	239 (12.7)	204 (14.9)	35 (6.8)
Bilirubin, μmol/L	10 [7, 13]	9 [7, 13]	10 [7, 14]
Bilirubin, not available	269 (14.3)	92 (6.7)	177 (34.2)
eGFR category			
≥90 mL/min/1.73 m^2^	679 (48.6)	425 (44.0)	254 (58.8)
≥60 and ≤89 mL/min/1.73 m^2^	569 (40.7)	410 (42.5)	159 (36.8)
≤59 mL/min/1.73 m^2^	149 (10.7)	130 (13.5)	19 (4.4)
Not available	488 (25.9)	402 (29.4)	86 (16.6)
Urea, mmol/L	4.9 [4.0, 6.1]	4.9 [3.9, 6.2]	4.9 [4.1, 6.0]
Urea, not available	183 (9.7)	119 (8.7)	64 (12.3)
Treatment regimens			
TDF	1,048 (55.6)	847 (62.0)	201 (38.8)
ETV	379 (20.1)	231 (16.9)	148 (28.6)
ETV + TDF	150 (8.0)	82 (6.0)	68 (13.1)
LAM/ADV + TDF	76 (4.0)	45 (3.3)	31 (6.0)
LAM/ADV + ETV	51 (2.7)	17 (1.2)	34 (6.6)
Other regimens	181 (9.6)	145 (10.6)	36 (6.9)

Data are the number (%) or median (IQR).

ADV, adefovir; ALT, alanine aminotransferase; ETV, entecavir; HBV, hepatitis B virus; IMD, Index of Multiple Deprivation; LAM, lamivudine; NA, nucleos(t)ide analogue; TDF, tenofovir disoproxil fumarate; VL, viral load.

### Distinct classes of virologic trajectories in CHB patients identify heterogeneity in response to NA therapy

**Fig. 1:**
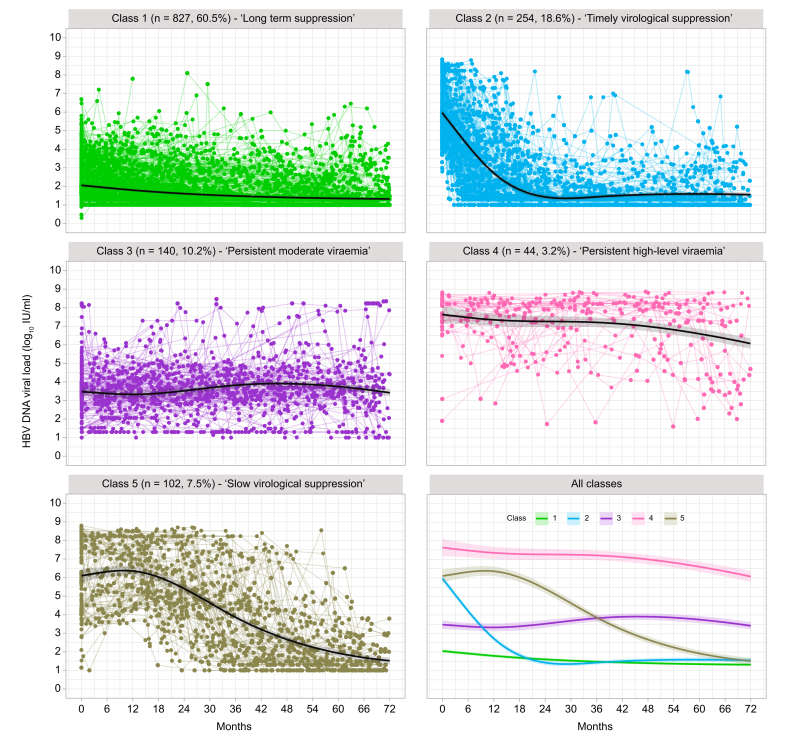
Individual trajectories of HBV VL for adults with chronic HBV infection on NA treatment divided into five classes using latent class mixed modelling. Data presented for derivation cohort (n = 1,367 individuals). Dots represent the raw values of VL linked by coloured lines for each individual, and solid black lines with shading area represent the predicted VL trajectory patterns with 95% CIs, which were computed by a Monte Carlo approximation of the posterior distribution of the predicted values. HBV, Hepatitis B Virus; NA, nucleos(t)ide analogue; VL, viral load.

An overview of virologic trajectories in all individual patients on NA therapy is shown in Fig. S2. With latent class mixed modelling in the derivation cohort, five mutually exclusive HBV DNA VL trajectories classes were identified (Fig. 1, Table S3), as follows:

• class 1 (n = 827, 60.5%) – ‘long-term suppression’;
• class 2 (n = 254, 18.6%) – ‘timely virological suppression’;
• class 3 (n = 140, 10.2%) – ‘persistent moderate viraemia’;
• class 4 (n = 44, 3.2%) – ‘persistent high-level viraemia’;
• class 5 (n = 102, 7.5%) – ‘slow virological suppression’.

The model showed a good discrimination of 0.93, with an entropy of 0.90, as well as high performance in classification with average posterior probability assignment for each class of 0.9509, 0.8543, 0.9221, 0.9436, and 0.9330 (Table S4). In the majority of individuals in class 2, VL suppressed to <50 IU/ml on treatment, which occurred at a median of 14 months (IQR 9–23 months) while in class 5 this level of suppression was only attained at a median of 51 months (IQR 39–63 months). The trajectory seen in patients with long-term viral suppression (class 1) is mostly likely to represent later time points of follow up of the same group as those with suppressed VL from higher baseline (class 2), so these two groups largely reflect the same clinical and biological response, but with data captured at different stages of the treatment journey.

Using the latent class mixed model estimated from the derivation cohort, we then classified VL trajectories of patients in the external validation cohort into the five defined classes, which also showed good discrimination of 0.93 and entropy of 0.88 (Table S4). The observed VL trajectories of the validation cohort against the identified five classes are illustrated in Fig. S3.

### Demographic and laboratory characteristics at baseline varied by VL class

**Fig. 2:**
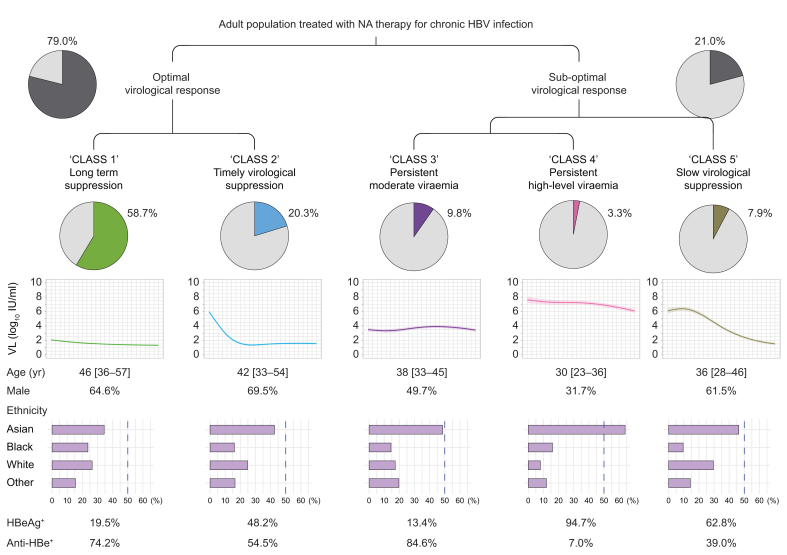
Schematic view of division of adult population with chronic HBV infection treated with NA therapy into five viral load classes. Data presented for 1,885 individuals by pooling the derivation and validation cohorts. Proportion of the population in each class and characteristics of each class are shown. Age in years is given as median [IQR]. Anti-HBe, Anti-Hepatitis B e-antigen; HBV, hepatitis B virus; HBeAg, Hepatitis B e-Antigen; NA, nucleos(t)ide analogue; VL, viral load.

Based on pooled data from derivation and validation cohorts, in 79.0% of longitudinal treatment episodes, there was an ‘optimal’ virological response (classes 1 and 2), while in 21% the virologic response was ‘sub-optimal’, that is, incomplete, non-sustained, or delayed (classes 3, 4, and 5) (Fig. 2).

Baseline characteristics stratified by VL classes are presented in Table 2 for the overall study cohort (and can be found in the Supplementary data for the derivation and validation cohorts separately, Table S5 and Table S6, respectively), showing that individuals who were classified into different VL classes had significantly different characteristics at baseline. The majority of those in classes 1, 2, and 5 were male (64.6%, 69.5%, and 61.5% respectively), whereas class 3 had a balanced distribution of both sexes, and class 4 predominantly consisted of females (68.3%), *p* <0.001 (Table 2). Classes 3, 4, and 5 were younger than classes 1 and 2 (*p* <0.001), which may suggest the treatment duration was shorter for these patients.

**Table 2:** Characteristics of patients at presentation stratified by the virologic trajectory patterns identified by latent class mixed modelling in the derivation cohort (n = 1,885).

Characteristics	Class 1 n = 1,106 (58.7%)	Class 2 n = 383 (20.3%)	Class 3 n = 185 (9.8%)	Class 4 n = 63 (3.3%)	Class 5 n = 148 (7.9%)	*p* value
	VL long-term suppressed	VL suppressed timely	VL persistent with moderate levels	VL persistent with high levels	VL suppressed slowly	
Sex, male	715 (64.6)	266 (69.5)	92 (49.7)	20 (31.7)	91 (61.5)	**<0.001**
Age, years	46 [36–57]	42 [33–54]	38 [33–45]	30 [23–36]	36 [28–46]	**<0.001**
Age group, years						**<0.001**
18–24	19 (1.7)	16 (4.2)	5 (2.7)	19 (30.2)	18 (12.2)	
25–34	213 (19.3)	99 (25.8)	59 (31.9)	25 (39.7)	46 (31.1)	
35–44	274 (24.8)	98 (25.6)	71 (38.4)	14 (22.2)	41 (27.7)	
45–54	259 (23.4)	84 (21.9)	39 (21.1)	3 (4.8)	24 (16.2)	
55–64	196 (17.7)	58 (15.1)	10 (5.4)	2 (3.2)	18 (12.2)	
65–74	111 (10.0)	18 (4.7)	1 (0.5)	0 (0.0)	1 (0.7)	
≥75	34 (3.1)	10 (2.6)	0 (0.0)	0 (0.0)	0 (0.0)	
Ethnic group						
Asian	318 (34.4)	132 (42.6)	84 (48.6)	32 (64.0)	58 (46.4)	**<0.001**
Black	219 (23.7)	50 (16.1)	25 (14.5)	8 (16.0)	12 (9.6)	
White	245 (26.5)	77 (24.8)	30 (17.3)	4 (8.0)	37 (29.6)	
Mixed/other ethnicity	143 (15.5)	51 (16.5)	34 (19.7)	6 (12.0)	18 (14.4)	
Not reported	181 (16.4)	73 (19.1)	12 (6.5)	13 (20.6)	23 (15.5)	
IMD (decile)	5 [3, 7]	5 [2, 7]	5 [3, 7]	4 [1, 7]	5 [2, 7]	0.425
IMD category						**0.030**
20% most deprived	225 (22.0)	96 (27.2)	29 (22.1)	20 (35.1)	45 (33.1)	
20% to 40%	277 (27.1)	69 (19.5)	28 (21.4)	10 (17.5)	21 (15.4)	
40% to 60%	223 (21.8)	76 (21.5)	29 (22.1)	12 (21.1)	35 (25.7)	
60% to 80%	177 (17.3)	63 (17.8)	27 (20.6)	9 (15.8)	17 (12.5)	
20% least deprived	122 (11.9)	49 (13.9)	18 (13.7)	6 (10.5)	18 (13.2)	
Not reported	82 (7.4)	30 (7.8)	54 (29.2)	6 (9.5)	12 (8.1)	
HBeAg status						
Negative	492 (80.5)	158 (51.8)	116 (86.6)	3 (5.3)	45 (37.2)	**<0.001**
Positive	119 (19.5)	147 (48.2)	18 (13.4)	54 (94.7)	76 (62.8)	
Not available	495 (44.8)	78 (20.4)	51 (27.6)	6 (9.5)	27 (18.2)	
Anti-HBe status						
Negative	176 (25.8)	138 (45.5)	21 (15.4)	53 (93.0)	72 (61.0)	**<0.001**
Positive	506 (74.2)	165 (54.5)	115 (84.6)	4 (7.0)	46 (39.0)	
Not available	424 (38.3)	80 (20.9)	49 (26.5)	6 (9.5)	30 (20.3)	
HBV VL, log_10_ IU/ml	1.5 [1.3, 2.6]	6.3 [5.1, 7.7]	3.7 [2.8, 4.5]	8.1 [7.5, 8.4]	6.3 [4.6, 8.2]	**<0.001**
HBV VL categories, IU/ml						**<0.001**
<20	164 (14.8)	0 (0.0)	1 (0.5)	0 (0.0)	1 (0.7)	
20 to <2,000	763 (69.0)	4 (1.0)	65 (35.1)	2 (3.2)	8 (5.4)	
2,000 to <20,000	121 (10.9)	29 (7.6)	68 (36.8)	0 (0.0)	13 (8.8)	
≥20,000	58 (5.2)	350 (91.4)	51 (27.6)	61 (96.8)	126 (85.1)	
ALT, IU/L	29 [20, 47]	64 [37, 125]	32 [21, 41]	35 [22, 50]	41 [32, 69]	**<0.001**
ALT, not available	41 (3.7)	24 (6.3)	21 (11.4)	12 (19.0)	25 (16.9)	
AST, IU/L	31 [24, 41]	45 [32, 74]	28 [23, 33]	27 [23, 57]	33 [27, 60]	**<0.001**
AST, not available	467 (42.2)	147 (38.4)	106 (57.3)	42 (66.7)	86 (58.1)	
Platelets, 10^9^/L	199 [156, 248]	201 [158, 238]	220 [170, 256]	206 [179, 244]	213 [179, 262]	**0.001**
Platelets, not available	83 (7.5)	54 (14.1)	40 (21.6)	25 (39.7)	45 (30.4)	
Albumin, g/L	40 [36, 43]	40 [37, 43]	43 [40, 46]	41 [37, 45]	41 [38, 44]	**<0.001**
Albumin, not available	81 (7.3)	53 (13.8)	35 (18.9)	25 (39.7)	42 (28.4)	
ALP, IU/L	79 [62, 101]	82 [63, 108]	69 [55, 96]	74 [61, 101]	75 [61, 102]	**0.005**
ALP, not available	81 (7.3)	53 (13.8)	35 (18.9)	25 (39.7)	45 (30.4)	
Bilirubin, μmol/L	10 [7, 13]	10 [7, 14]	9 [6, 12]	8 [6, 11]	10 [7, 13]	**0.001**
Bilirubin, not available	109 (9.9)	60 (15.7)	38 (20.5)	20 (31.7)	42 (28.4)	
eGFR categories, ml/min/1.73 m^2^						**<0.001**
≥90	397 (45.6)	147 (51.6)	66 (51.2)	17 (77.3)	52 (57.1)	
≥60 and ≤89	350 (40.2)	125 (43.9)	54 (41.9)	5 (22.7)	35 (38.5)	
≤59	123 (14.1)	13 (4.6)	9 (7.0)	0 (0.0)	4 (4.4)	
Not available	236 (21.3)	98 (25.6)	56 (30.3)	41 (65.1)	57 (38.5)	
Urea, mmol/L	5.0 [4.0, 6.4]	4.8 [4.0, 5.8]	4.6 [3.9, 5.8]	4.0 [3.3, 4.6]	5.0 [4.1, 5.9]	**<0.001**
Urea, not available	61 (5.5)	29 (7.6)	38 (20.5)	19 (30.1)	36 (24.3)	
Treatment regimens						**<0.001**
TDF	562 (50.8)	253 (66.1)	115 (62.2)	40 (63.5)	78 (52.7)	
ETV	234 (21.2)	68 (17.8)	40 (21.6)	8 (12.7)	29 (19.6)	
ETV + TDF	64 (5.8)	38 (9.9)	17 (9.2)	9 (14.3)	22 (14.9)	
LAM/ADV + TDF	58 (5.2)	8 (2.1)	3 (1.6)	1 (1.6)	6 (4.1)	
LAM/ADV + ETV	47 (4.2)	1 (0.3)	2 (1.1)	0 (0.0)	1 (0.7)	
Other regimens	141 (12.7)	15 (3.9)	8 (4.3)	5 (7.9)	12 (8.1)	

Data are the number (%) or median [IQR]. Comparison was conducted across non-missing categories for a categorical variable. For categorical variables, Χ^2^ or Fisher’s exact tests were used for comparison. For continuous variables, *t* or Wilcoxon tests were used for two groups comparison, whilst ANOVA or the Kruskal–Wallis test was used for more than two groups comparison. All significance tests performed were two-sided.

Bold indicates *p* value lower than 0.05.

ADV, adefovir; ALT, alanine aminotransferase; Anti-HBe, Anti-Hepatitis B e-antigen; ETV, entecavir; HBeAg, Hepatitis B e-Antigen; IMD, Index of Multiple Deprivation; LAM, lamivudine; TDF, tenofovir disoproxil fumarate; VL, viral load.

Class 4 contained a higher proportion of individuals of Asian ancestry (64.0%) compared with other groups (34.4% to 48.6%, *p* <0.001). Most patients in class 3 (VL persistent at moderate levels) were anti-HBe positive (84.6%), while 94.7% in class 4 were HBeAg positive at baseline, perhaps representing the infection phenotype sometimes termed ‘immunotolerant’, which may account for differences in VL levels. Patients in class 2 had a significantly higher median ALT level (median 64, [IQR 37–125] IU/L) at baseline, followed by class 5 (median 41, [IQR 32–69] IU/L), compared with other classes (median 29, [IQR 20–47] IU/L for class 1; median 32 [IQR 21–41] IU/L for class 3; median 35 [IQR 22–50] IU/L for class 4), *p* <0.001.

The majority of patients in each class received monotherapy with TDF (between 51%-66% in all classes), followed by monotherapy with ETV (13%-22%), Table 2. A higher proportion of individuals in classes 4 and 5 received a combination of TDF and ETV (14.3% and 14.9% in these two classes vs 5.8%, 9.9% and 9.2% in other three classes, *p* <0.001), likely reflecting clinical decisions aligned with treatment guidelines to add a second agent when VL is not suppressed over time (7,17) (Table 2).

### ALT decreased over time in classes with suppressed VL

We assessed longitudinal trends of ALT by linear mixed effects models (considering repeated measurements, and adjusted for age, sex, and ethnicity). ALT decreased at differing rates over time in the VL suppressed groups (classes 1, 2, and 5; all *p* <0.001), in line with declining VL trajectories of these classes, whereas ALT levels of patients in classes 3 and 4 (with persistent viraemia) did not significantly decrease over time or worsened (Fig. S4, Table 3). In class 1, ALT levels continuously decreased but only marginally so (coefficient: -0.0026, 95% CI: -0.0034 to -0.0018), as most of the patients already had normalised ALT at baseline compared with other classes (Fig. S5).

**Table 3:** Longitudinal trend of ALT over time for distinct virologic trajectory patterns for patients with chronic HBV infection on NA therapy in the overall study cohort.

	Intercept (95% CI), log IU/L	Coefficient β (95% CI)
Class 1 (VL long-term suppressed)	3.95 (3.85, 4.05)∗∗∗	-0.0026 (-0.0034, -0.0018)∗∗∗
Class 2 (VL suppressed timely)	4.39 (4.29, 4.50)∗∗∗	-0.0108 (-0.0122, -0.0094)∗∗∗
Class 3 (VL persistent with moderate levels)	4.01 (3.88, 4.13)∗∗∗	-0.0007 (-0.0026, 0.0012)
Class 4 (VL persistent with high levels)	4.13 (3.96, 4.30)∗∗∗	-0.0006 (-0.0037, 0.0026)
Class 5 (VL suppressed slowly)	4.48 (4.35, 4.60)∗∗∗	-0.0085 (-0.0106, -0.0065)∗∗∗

Derivation and validation cohorts were combined according to the VL trajectories. 1,883 patients (out of 1,885, 99.89%) had longitudinal measurements of ALT for modelling. The longitudinal trends of ALT over time were assessed by linear mixed effects models with random intercepts and slopes. All linear mixed effects models were adjusted for demographics (including age, sex, and ethnicity). ∗∗∗*p* <0.001 (Wald test).

ALT, alanine aminotransferase; HBV, hepatitis B virus; NA, nucleos(t)ide analogue; VL, viral load.

We compared changes in ALT in class 5 compared to class 2, because they had similar baseline ALT and VL levels. ALT levels in class 5 decreased more slowly compared with class 2 (coefficient: -0.0108, 95% CI: -0.0122 to -0.0094 for class 2 *vs.* coefficient: -0.0085, 95% CI: -0.0106 to -0.0065 for class 5, Table 3, Fig. S4). Varying proportions of patients had elevated ALT over time among different classes (Figs. S5 and S6). Class 1 maintained similar ALT levels over time, class 2 experienced timely improvements of ALT levels, and class 5 showed a slower improvement, in keeping with VL suppression trajectories.

### Risk of liver fibrosis/cirrhosis varied by VL class

For disease progression analysis, we analysed 1,412 individuals for whom data were available on fibrosis/cirrhosis status, combining derivation and validation cohorts. The majority (53%) of the fibrosis/cirrhosis cases were identified by biopsy, elastography, imaging, or ICD data, the remainder were identified by FIB-4 or APRI scores (Table S7). Overall, those with liver fibrosis or cirrhosis compared with those without were more likely to be male (71.4% *vs.* 58.4%) and older (46 years *vs.* 40 years) (*p* <0.001). Individuals in class 5 (who suppressed VL slowly) were more likely to have liver fibrosis/cirrhosis compared with other classes (*p* <0.001) (Table 4).

**Table 4:** **Baseline characteristics of patients with *vs.* without the presence of liver fibrosis and/or cirrhosis in the overall cohorts, combining derivation and validation cohorts according to the VL trajectory classes.**.

Parameter	Overall	No presence of liver fibrosis and cirrhosis	Presence of liver fibrosis or cirrhosis	*p* value
Number of patients	1,412	1,080	332	
VL trajectory pattern				**<0.001**
Class 1	797 (56.4)	611 (56.6)	186 (56.0)	
Class 2	268 (19.0)	204 (18.9)	64 (19.3)	
Class 3	167 (11.8)	143 (13.2)	24 (7.2)	
Class 4	58 (4.1)	46 (4.3)	12 (3.6)	
Class 5	122 (8.6)	76 (7.0)	46 (13.9)	
Sex, male	868 (61.5)	631 (58.4)	237 (71.4)	**<0.001**
Age, years	41 [33–52]	40 [32–50]	46 [36–57]	**<0.001**
Age group, years				**<0.001**
18-24	63 (4.5)	48 (4.4)	15 (4.5)	
25-34	359 (25.4)	300 (27.8)	59 (17.8)	
35-44	396 (28.0)	321 (29.7)	75 (22.6)	
45-54	307 (21.7)	232 (21.5)	75 (22.6)	
55-64	193 (13.7)	126 (11.7)	67 (20.2)	
65-74	71 (5.0)	41 (3.8)	30 (9.0)	
≥75	23 (1.6)	12 (1.1)	11 (3.3)	
Ethnic group				0.15
Asian	511 (43.1)	403 (44.8)	108 (37.8)	
Black	216 (18.2)	154 (17.1)	62 (21.7)	
White	266 (22.4)	199 (22.1)	67 (23.4)	
Mixed/other ethnicity	193 (16.3)	144 (16.0)	49 (17.1)	
Not reported	226 (16.0)	180 (16.7)	46 (13.9)	
IMD				0.78
20% most deprived	340 (26.9)	259 (27.3)	81 (25.7)	
20% to 40%	291 (23.0)	220 (23.2)	71 (22.5)	
40% to 60%	268 (21.2)	205 (21.6)	63 (20.0)	
60% to 80%	206 (16.3)	151 (15.9)	55 (17.5)	
20% least deprived	160 (12.6)	115 (12.1)	45 (14.3)	
Not reported	147 (10.4)	130 (12.0)	17 (5.1)	
Coinfection (Yes)	57 (4.0)	37 (3.4)	20 (6.0)	0.05
HBeAg status				0.38
Negative	675 (68.6)	521 (67.8)	154 (71.3)	
Positive	309 (31.4)	247 (32.2)	62 (28.7)	
Not available	428 (30.3)	312 (28.9)	116 (34.9)	
Anti-HBe status				0.43
Negative	340 (34.3)	269 (35.0)	71 (31.8)	
Positive	652 (65.7)	500 (65.0)	152 (68.2)	
Not available	420 (29.7)	311 (28.8)	109 (32.8)	
HBV VL categories, IU/ml				0.18
<20	97 (6.9)	76 (7.0)	21 (6.3)	
20 - <2,000	637 (45.1)	477 (44.2)	160 (48.2)	
2,000 - <20,000	193 (13.7)	159 (14.7)	34 (10.2)	
≥20,000	485 (34.3)	368 (34.1)	117 (35.2)	
ALT, log IU/L	3.5 [3.1, 3.9]	3.5 [3.0, 3.9]	3.6 [3.2, 4.1]	**<0.001**
ALP, IU/L	75 [60, 95]	72 [59, 91]	83 [64, 114]	**<0.001**
Albumin, g/L	41 [38, 44]	41 [39, 44]	40 [36, 43]	**<0.001**
AST, IU/L	30 [24, 40]	29 [24, 37]	35 [28, 49]	**<0.001**
Platelets, 10^9^/L	209 [171, 250]	216 [182, 257]	186 [138, 228]	**<0.001**
Bilirubin, μmol/L	9 [7, 13]	9 [6, 12]	11 [8, 15]	**<0.001**
eGFR categories, ml/min/1.73 m^2^				**<0.001**
≥90	506 (48.4)	392 (50.4)	114 (42.5)	
≥60 and ≤89	448 (42.8)	340 (43.7)	108 (40.3)	
≤59	92 (8.8)	46 (5.9)	46 (17.2)	
Not available	366 (25.9)	302 (28.0)	64 (19.3)	
Urea, mmol/L	4.9 [4.0, 6.0]	4.8 [3.9, 5.9]	4.9 [4.1, 6.3]	**0.02**
Treatment regimens				**<0.001**
TDF	836 (59.2)	671 (62.1)	165 (49.7)	
ETV	282 (20.0)	215 (19.9)	67 (20.2)	
ETV + TDF	111 (7.9)	70 (6.5)	41 (12.3)	
LAM/ADV + TDF	46 (3.3)	30 (2.8)	16 (4.8)	
LAM/ADV + ETV	29 (2.1)	18 (1.7)	11 (3.3)	
Other regimens	108 (7.6)	76 (7.0)	32 (9.6)	

A total of 1,412 patients with data available for determining liver fibrosis and cirrhosis were included for the comparison. Data are the number (%) or median [IQR]. Comparison was conducted across non-missing categories for a categorical variable. For categorical variables, Χ^2^ or Fisher’s exact tests were used for comparison. For continuous variables, *t* or Wilcoxon tests were used for two groups comparison, whilst ANOVA or the Kruskal–Wallis test was used for more than two groups comparison. All significance tests performed were two-sided.

Bold indicates *p* value lower than 0.05.

ADV, adefovir; ALP, alkaline phosphatase; ALT, alanine aminotransferase; Anti-HBe, Anti-Hepatitis B e-antigen; AST, aspartate aminotransferase; eGFR, estimated glomerular filtration rate; ETV, entecavir; HBV, hepatitis B virus; HBeAg, Hepatitis B e-Antigen; IMD, Index of Multiple Deprivation; IQR, interquartile range; LAM, lamivudine; TDF, tenofovir disoproxil fumarate; VL, viral load.

K–M analysis demonstrated that individuals in class 5 (slow VL suppression) were more likely to have liver fibrosis/cirrhosis over follow up to 108 months, compared with class 1 (*p* = 0.003, Fig. S7). The cumulative probability of being free of fibrosis/cirrhosis was similar between class 1 and class 2 (*p* = 0.73), as well as between classes 3 and class 4 (*p* = 0.47) (Fig. S7). Individuals in class 3 were less likely to have liver fibrosis and cirrhosis at baseline compared to class 1, but the cumulative probability of being free of fibrosis/cirrhosis for class 3 decreased rapidly over time, which may reflect increasing age (Fig. S7). A similar trend was observed for class 4 (Fig. S7, Table S8). K–M curves were constructed without adjustment for confounders, so other attributes of the class (age, sex, HBeAg status) may be driving the observed differences.

**Fig. 3:**
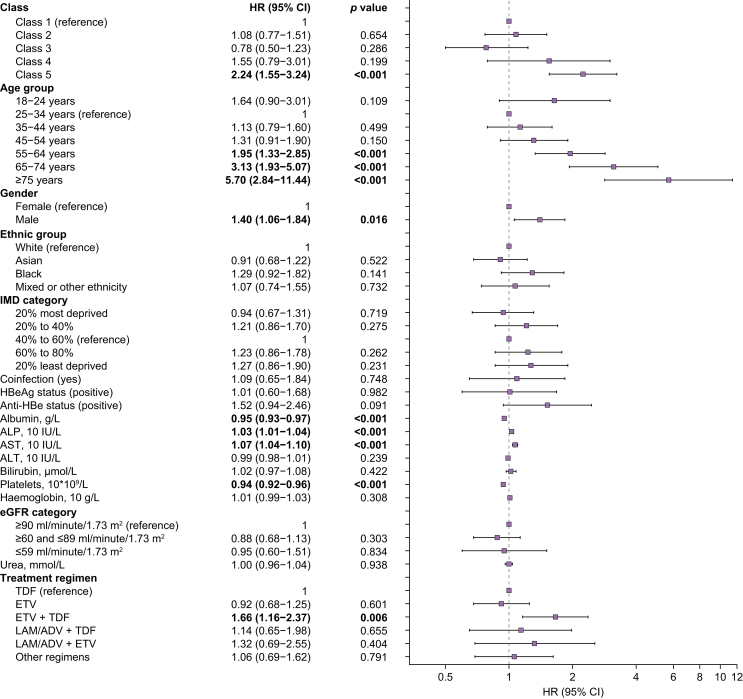
Multivariate Cox proportional-hazards model investigating associations of different VL trajectory patterns with liver disease progression to fibrosis and/or cirrhosis among adults with chronic HBV infection on NA therapy. Forest plot showing the hazard ratios and 95% CIs for the development of liver fibrosis and cirrhosis, which were calculated with Cox proportional-hazards model. ADV, adefovir; ALP, alkaline phosphatase; ALT, alanine transaminase; Anti-HBe, Anti-Hepatitis B e-antigen; AST, aspartate aminotransferase; eGFR, estimated glomerular filtration rate; ETV, entecavir; HBV, hepatitis B virus; HBeAg, Hepatitis B e-Antigen; HR, hazard ratio; IMD, Index of Multiple Deprivation; LAM, lamivudine; NA, nucleos(t)ide analogue; TDF, tenofovir disoproxil fumarate; VL, viral load.

Multivariate analysis (fully adjusted for demographics, laboratory parameters, and treatment regimens) showed that patients in class 5 had ∼2-fold increased hazards of progression to fibrosis/cirrhosis compared with those patients with VL long-term suppressed (class 1) (adjusted hazard ratio [HR], 2.24; 95% CI, 1.55–3.24) (Fig. 3, Table S9). Compared with those patients aged 25–34 years, those aged 55–64 years, 65–74 years, and ≥75 years had ∼2-fold (adjusted HR 1.95; 95% CI, 1.33–2.85), ∼3-fold (adjusted HR, 3.13; 95% CI, 1.93–5.07), and ∼6-fold (adjusted HR, 5.70; 95% CI, 2.84–11.44) increased hazards of progression to liver fibrosis/cirrhosis, respectively, while males had 1.40-fold increased hazards of progression (adjusted HR, 1.40; 95% CI, 1.06–1.84) compared with females. As expected, lower albumin and platelets, and higher AST and ALP, were significantly associated with fibrosis/cirrhosis (Fig. 3, Table S9). Sensitivity analysis only adjusting for age and sex, shows similar hazards ratio for each VL trajectory class (Table S10).

**Fig. 4:**
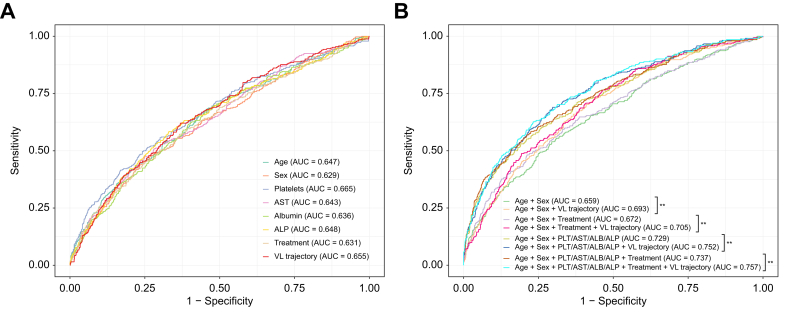
ROC curves of HBV VL trajectory and other identified predictors for liver fibrosis and cirrhosis. (A) Single identified predictor; (B) combination of different identified predictors. All models were adjusted for other parameters that were included in multivariate analysis. In panel B, ∗∗*p* <0.01 using the roc.test function with the DeLong’s test method. ALB, albumin; ALP, alkaline phosphatase; AST, aspartate aminotransferase; HBV, hepatitis B virus; PLT, platelets; NA, nucleos(t)ide analogue; ROC, receiver operating characteristic curve; VL, viral load.

ROC analyses demonstrated that the predictive ability of VL trajectory for liver fibrosis and cirrhosis (AUC = 0.66) was comparable to that of other well-known predictors in previous studies, such as age (AUC = 0.65), sex (AUC = 0.63), and platelets (0.67) (Fig. 4A; all pairwise *p* values non-significant, Table S11). Furthermore, we demonstrated the addition of VL trajectory into the combinations of other predictors for liver fibrosis and cirrhosis significantly improved the prediction performance (Fig. 4B, Table S12, all *p* values <0.01), which can further improve early identification of patients at risk of liver complications.

### Sensitivity and subgroup analyses

Sensitivity analysis excluding NA agents other than TDF and ETV shows similar hazard ratios for each VL trajectory class as the primary analysis (Table S13). We also performed subgroup analyses on classes 3, 4, and 5, to further evaluate potentially relevant markers.

For class 3, most individuals were HBeAg negative (116/185, 86.6%). In the HBeAg-negative subgroup (n = 116), analysis did not find a significant HR for clinical progression to fibrosis/cirrhosis in patients with baseline HBV DNA ≥2,000 IU/ml (crude HR: 0.52; 95% CI: 0.23–1.16, *p* = 0.646) or ≥20,000 IU/ml (crude HR: 1.19; 95% CI: 0.37–3.81, *p* = 0.765).

For class 4, we performed subgroup analysis to explore if ALT level at the start of therapy is associated with outcome. Univariate analysis showed a relationship between higher baseline ALT and progression to liver fibrosis/cirrhosis (*p* = 0.02); however, this association was not significant in the multivariable analysis after adjusting for confounders including age, sex, ethnicity, and deprivation scores.

For class 5, among those individuals receiving two agents in combination (TDF and ETV), 14 had VL measurements available before and after switching to dual therapy (the VL measurement closest to the switch was used). In this group, nine of 14 (64%) had a decline of VL, with mean of decline of 1.6 log_10_ IU/ml.

Two study sites measured quantitative HBsAg (qHBsAg) levels in their clinical practice over the period of data collection. Thus, qHBsAg data were available for 329/1,885 individuals (17.5%), with longitudinal data available for 273/1,885 (14.5%), providing a total of 1,072 qHBsAg measurements (median of three measurements per patient [IQR 3–5, range 2–12]). We assessed longitudinal trends of qHBsAg levels using linear mixed effects models, adjusted for age, sex, and ethnicity (Table S14). qHBsAg decreased over time in groups in which VL suppressed (classes 1, 2, and 5; all *p* <0.01), with qHBsAg levels in class 2 decreasing at the highest rate (coefficient: -0.0163, 95% CI -0.0217 to -0.0108 for class 2 *vs.* coefficient -0.0102, 95% CI -0.0174 to -0.0032 for class 5 *vs.* coefficient -0.0084, 95% CI -0.0116 to -0.0052 for class 1). In contrast, qHBsAg in classes 3 and 4 (with persistent viraemia) did not significantly decrease. For classes 1, 2, and 3, the proportion of patients with qHBsAg <100 IU/ml increased in the most recent measurements compared with the initial measurements, whilst this trend was not observed for classes 4 and 5 (Table S15).

### Minimum number of repeated measurements of VL required to assign class

We conducted sensitivity analysis on the validation cohort, to ascertain how robustly individuals can be classified into one of our five classes with a limited number of repeated VL measurements. Using only the first two VL measurements per individual (but retaining the requirement for ≥6 months follow-up measurements of HBV DNA VL in line with our original eligibility criteria), the classification still achieved a good discrimination (0.83) and entropy (0.73). These data spanned a median follow-up duration of 12 months (IQR 7–19) (Table S16).

We repeated this analysis, but this time removing restrictions on the minimum follow-up duration for each patient, which demonstrated that three or more VL measurements are required for assigning an individual into one of our five classes with good discrimination (0.82) and entropy (0.72) (Table S17). These three measurements spanned a median of 12 months follow up (IQR 6–21), similar to that in the above scenario. Therefore, individuals with two or three repeated measurements of VL spanning a 1-year period can be reliably assigned into a class based on this model.

### Relationship between VL classes and EASL definition of virological breakthrough

Based on definitions in EASL guidelines,^7^ we determined the proportion of individuals in our overall cohort, and in each of the five classes, who experienced virological breakthrough on NA treatment. We found breakthrough in 6.4% (120/1,885), and the proportion in classes 1–5 was 4.0%, 2.9%, 17.3%, 30.2%, and 9.5%, respectively (Table S18). Among the 120 individuals with virological breakthrough, 84 had specific data on longitudinal therapy; in 25/84, treatment strategy was switching to another drug, or from a single drug to a combination therapy.

## Discussion

### Summary of novelty and key findings

The main goal and novelty of this study is to describe and classify VL trajectories in a real-world dataset representing chronic HBV infection, to develop a better understanding of treatment outcomes at a population level. Recognising that many more people worldwide will become eligible for HBV therapy as treatment recommendations change,^19^ these data are highly relevant to the clinical and public health landscape. We identified and validated five novel and distinct VL trajectory classes using an unbiased discovery method, showing that these classes are characterised by distinct clinical, laboratory, and demographic characteristics. In particular, the relationship between VL trajectories and ALT provides evidence of the clinical significance of this classification.

These real-world data are an important addition to our understanding of treatment responses, in contrast to much existing HBV treatment data which are derived from closely monitored and highly selected cohorts taking part in clinical trials. We are thus uniquely positioned to determine the outcomes of treatment across wide populations where diverse and complex factors operate to influence outcomes, for example including comorbidity, polypharmacy, alcohol use, socioeconomic factors, and varied adherence to therapy. To our knowledge, this is the first study to comprehensively document and classify long-term virologic trajectories in a large and unselected population on HBV treatment, and to explore VL trajectory subcohorts/classes where virological response to treatment may be suboptimal. Understanding VL suppression has implications for both individual disease progression and also HBV outcomes and transmission at a population level. As guidelines are moving towards relaxed eligibility criteria for treatment, there is a heightened need to understand responses and outcomes of therapy, and to provide resources and an evidence base to intervene when an adequate virologic response is not achieved.

In the majority of individuals (∼80%) HBV VL suppressed on treatment as expected, assigned as classes 1 and 2. However, importantly, we observed suboptimal virologic responses in ∼20% of individuals treated with NA agents for CHB, with persistent moderate or high VL (classes 3 and 4), or slow suppression of VL (class 5), which may have significant implications for clinical outcomes. Documenting and classifying these distinct on-treatment phenotypes can allow discrimination between satisfactory virologic treatment responses, *vs.* situations in which a suboptimal response might expose an individual to excess clinical risks. In the latter situation, patients may benefit from enhanced monitoring, interventions to help optimise adherence (e.g. education, peer support, review of comorbid conditions), and/or alterations to therapy. At a public health level, quantifying the risks of non-suppression of viraemia on NA treatment is important to allow modelling of population outcomes as increased numbers of people will be diagnosed and treatment eligible over the years ahead. For example, many new HBV diagnoses are being made as a result of the NHS England programme to implement routine ‘opt-out’ testing in Emergency Department attenders.^35^

### Relationship between VL class and clinical outcome

Individuals in class 5 (slow suppression of VL) had an increased risk of liver fibrosis/cirrhosis within the observation period, compared with class 1. However, we did not find a significant association between non-suppressing groups (classes 3 and 4) and the risk of liver fibrosis/cirrhosis, potentially reflecting the younger age of these groups and enrichment for female sex, which is known to be protective,^36^ compared with class 1. In keeping with this, the lower hazards of liver fibrosis/cirrhosis in class 3 were not significant and class 4 (persistent high viraemia) is close to significance after adjustment for the relevant confounders. In addition, the K–M analysis shows that although patients in classes 3 and 4 at the early time points of observation were less likely to develop liver fibrosis/cirrhosis compared to class 1, they progressed to liver fibrosis/cirrhosis rapidly over time with increasing age. This highlights the need for longer follow up to determine associations with disease progression, which may only become apparent in older adults. Furthermore, increased sample size for classes 3 and 4 is needed to support further investigation into the association with liver disease progression.

After correction for confounders, only class 5 retained a significant relationship with the development of fibrosis/cirrhosis. This observation is not simply related to VL at baseline, as classes 2, 4, and 5 all started their trajectory with high VL, and univariate analysis showed that baseline VL was not significantly associated with disease progression (Table S8). We also conducted sensitivity analyses with a simpler model only adjusting for age and sex or excluding the old NA agents, showing similar results (class 5 associated with liver fibrosis/cirrhosis).

To determine the applicability of this approach to other real-world data, we evaluated the performance of the model using smaller number of VL measurements per patient, which demonstrated three VL measurements are required without restriction on minimum follow-up duration, or two VL measurements at least 6 months apart, to discriminate between classes. Our model can thus be considered for predicting outcomes in different settings:

(i) to identify individuals who are at high risk of non-suppression on NA treatment, and thus may warrant closer scrutiny to avoid complications;
(ii) to improve the early prediction of liver fibrosis and cirrhosis, by incorporating VL trajectory classifications into existing scoring tools;
(iii) to inform preventive interventions, exemplified by antiviral prophylaxis for the prevention of mother to child transmission, when a consistent and timely reduction in viraemia is required;
(iv) to inform public health and programmatic interventions which seek to deliver on population elimination targets.

### Caveats and limitations

Our analysis was limited by the missingness of certain data. For example, we were unable to adjust for metabolic laboratory parameters such as total cholesterol, triglycerides and glucose, although emerging evidence suggests the combination of metabolic disease and CHB can accelerate the progression of liver pathology.^37^^,^^38^ As body mass index and alcohol were missing for majority of the study subjects in this study, we were unable to consider these risk factors in the analysis for the development of liver fibrosis and cirrhosis. qHBsAg levels are important for predicting immunological control and functional cure, however, qHBsAg was not routinely tested at all sites. Therefore, we analysed qHBsAg levels in only a small subset of the cohort. There are limitations in the relationship between laboratory and imaging scores of fibrosis (elastography, APRI, FIB-4) and underlying histological liver disease. However, as biopsy is not widely undertaken these tools are now the standard of care for clinical assessment and decision-making. HCC incidence was not robustly reported and the number of HCC cases was insufficient for analysis.

The dataset does not contain details of the potential underlying causes for non-suppression of viraemia, which include treatment interruptions or incomplete adherence to therapy, inadequate drug levels, and genotypic viral resistance; further work is needed to dissect these causes and to understand their relative contributions. Our virologic groups partly overlap with classification presented in EASL guidelines, as we were able to identify individuals with virological breakthrough based on existing definitions. However, lack of robust treatment histories that include a documented treatment start date meant we could not reliably assign individuals to other EASL classes.

### Further work

Longer periods of follow up in large datasets are required to develop a better understanding of the association between non-suppressed VL patterns and outcomes, particularly risks of cirrhosis, HCC and transmission events. Prospectively, data collection through NIHR HIC network will continue, and the quality and completeness of imaging data and electronic prescribing data will improve, providing us with more power to identify significant long-term outcomes. Promisingly, with growing interest in HBsAg dynamics as a marker of functional cure, this measurement will become more widely available. Patient feedback from our Patient and Public Involvement activities, is supportive of collecting these data. The rationale for – and outcomes of – dual antiviral therapy will also be more thoroughly explored in future analyses.

### Conclusions

There is significant heterogeneity in virologic response to HBV NA treatment. Complete virologic suppression on NA treatment can be slow or incomplete, correlating in some cases with enhanced clinical risk. Enhanced understanding of treatment response can be used to inform better risk-stratification, improved patient-centric clinical care, models of treatment response at a population level, and as a foundation to understand the additional impact of novel therapies as these become available. Additional scrutiny is needed to understand the determinants of non-suppression so that social, clinical and biological risk factors can be tackled. Optimising use of NA therapy is fundamental to reducing morbidity and mortality, and decreasing transmission, which are crucial attributes of international targets to eliminate HBV infection as a public health threat.

## Abbreviations

ADV, adefovir; AIC, Akaike information criteria; ALP, alkaline phosphatase; ALT, alanine transaminase; Anti-HBe, Anti-Hepatitis B e-antigen; APRI, aspartate aminotransferase to platelet ratio index; AST, aspartate aminotransferase; BIC, Bayesian information criteria; CHB, chronic hepatitis B; CUH, Cambridge University Hospitals NHS Foundation Trust; eGFR, estimated glomerular filtration rate; EPR, electronic patient record; ETV, entecavir; FIB-4, Fibrosis-4; HBeAg, Hepatitis B e-Antigen; HCC, hepatocellular carcinoma; HIC, Health Informatics Collaborative; HR, hazard ratio; ICD, International Classification of Diseases; ICHT, Imperial College Healthcare NHS Trust; IMD, Index of Multiple Deprivation; K–M, Kaplan–Meier; LAM, lamivudine; LUH, Liverpool University Hospitals NHS Foundation Trust; NA, nucleos(t)ide analogue; NIHR, National Institute for Health and Care Research; OUH, Oxford University Hospitals NHS Foundation Trust; qHBsAg, quantitative HBsAg; ROC, receiver operating characteristic; TAF, tenofovir alafenamide; TDF, tenofovir disoproxil fumarate; UCLH, University College London Hospitals NHS Foundation Trust; UHS, University Hospital Southampton NHS Foundation Trust; ULN, upper limit of normal; VL, viral load; WHO, World Health Organization.

## Financial support

This work has been conducted using National Institute for Health and Care Research Health Informatics Collaborative (NIHR HIC) data resources and funded by the NIHR HIC and has been supported by NIHR Biomedical Research Centres at Cambridge, Imperial, Oxford, Southampton, and University College London Hospitals (UCLH). GSC is an NIHR research professor, EB is an NIHR senior investigator. PCM is funded by the Wellcome Trust (ref. 110110), the Francis Crick Institute (ref CC2223), and UCLH NIHR Biomedical Research Centre. CC received partial doctoral funding from GlaxoSmithKline (GSK) at the University of Oxford. AJS is supported by a Senior Clinical Lectureship from the NIHR at the University of Liverpool. The views expressed in this article are those of the authors and not necessarily those of the National Health Service, the NIHR or the Department of Health.

## Authors’ contributions

Contributed to the conception and design of the work: EB, PCM, TW. Directed the data collation and implementation of the study: EB, PCM, GSC, WG, SIK, EN, KW, JD. Contributed to the methodology development and/or the data acquisition, processing, interpretation, and management of study data, with the support from the NIHR HIC Viral Hepatitis and Liver Disease Consortium: TW, CC, AJS, ST, KM, AF, JJ, BG, DP, LM, EM, CRJ, HS, GR, SL, TN, KAV, CD, AIH, MG, FB, LE, LR, DR. Designed the analytical strategy: TW, PCM. Conducted the analysis: TW. Supervision: EB, PCM. Helped interpret the findings: PCM, EB, AJS, WG, SIK, GSC, EN. Wrote the original draft: TW and PCM. Reviewed and revised the manuscript critically and approved the final version for publication: all authors.

## Data availability statement

The NIHR HIC Data Sharing Framework provides an overarching governance approach for data sharing between NHS organisations for research purposes. Research proposals are reviewed by the relevant NIHR HIC Scientific Steering Committees and in line with the NIHR HIC Data Sharing Framework. If you are interested in finding out more about access to datasets within NIHR HIC data collaborations please see our website for further information - https://hic.nihr.ac.uk/. Code is available on Figshare under the following https://doi.org/10.6084/m9.figshare.27094348.

## Ethics approval

The research database for the NIHR HIC Viral Hepatitis and Liver Disease theme was approved by South Central—Oxford C Research Ethics Committee (REF Number: 21/SC/0060). All methods for data collection, transmission, and management for this study were carried out in accordance with relevant guidelines and regulations. The requirement for written informed consent was waived by South Central—Oxford C Research Ethics Committee, because data have been in effect anonymised before transfer to the research database.

## Conflicts of interest

GC reports personal fees from Gilead and Merck Sharp & Dohme, outside the submitted work. ST reports she has previously received Gilead Investigator-led grant for a viral hepatitis project. WG reports personal fees from GSK outside the submitted work. EB has consulted for, and received research grants from Roche and GSK. EB and PCM have academic collaborative partnerships with GSK. Other authors have no conflict of interest.

Please refer to the accompanying ICMJE disclosure forms for further details.
