# Supplementary material for: Distinct virologic trajectories in chronic hepatitis B identify heterogeneity in response to nucleos(t)ide analogue therapy
Source: JHEP Rep. 2024 Oct 9;7(1):101229. doi: 10.1016/j.jhepr.2024.101229 (PMC11664071; doi:10.1016/j.jhepr.2024.101229)
Supplement: Multimedia component 1 [file mmc1.pdf]

Distinct virologic trajectories in chronic hepatitis B identify heterogeneity in response to nucleos(t)ide analogue therapy

Tingyan Wang, Cori Campbell, Alexander J Stockdale, Stacy Todd, Karl McIntyre, Andrew Frankland, Jakub Jaworski, Ben Glampson, Dimitri Papadimitriou, Luca Mercuri, Erik Mayer, Christopher R Jones, Hizni Salih, Gail Roadknight, Stephanie Little, Theresa Noble, Kinga A Várnai, Cai Davis, Ashley I Heinson, Michael George, Florina Borca, Louise English, Luis Romão, David Ramlakhan, NIHR HIC Viral Hepatitis and Liver Disease Consortium, Kerrie Woods, Jim Davies, Eleni Nastouli, Salim I Khakoo, William Gelson, Graham S Cooke, Eleanor Barnes, Philippa C Matthews

Table of contents

Fig. S1……………………………………………………………………………………3

Fig. S2……………………………………………………………………………………4

Fig. S3……………………………………………………………………………………5

Fig. S4……………………………………………………………………………………6

Fig. S5……………………………………………………………………………………7

Fig. S6……………………………………………………………………………………8

Fig. S7……………………………………………………………………………………9

Table S1………………………………………………………………………………...10

Table S2………………………………………………………………………………...11

Table S3………………………………………………………………………………...12

Table S4………………………………………………………………………………...12

Table S5………………………………………………………………………………...13

Table S6……………………………………………………………………………...16

Table S7……………………………………………………………………………...20

Table S8……………………………………………………………………………...20

Table S9……………………………………………………………………………...21

Table S10…………………………………………………………………………....23

Table S11…………………………………………………………………………....24

Table S12…………………………………………………………………………....24

Table S13…………………………………………………………………………....25

Table S14…………………………………………………………………………....27

Table S15…………………………………………………………………………....28

Table S16…………………………………………………………………………....29

Table S17…………………………………………………………………………....30

Table S18…………………………………………………………………………....31

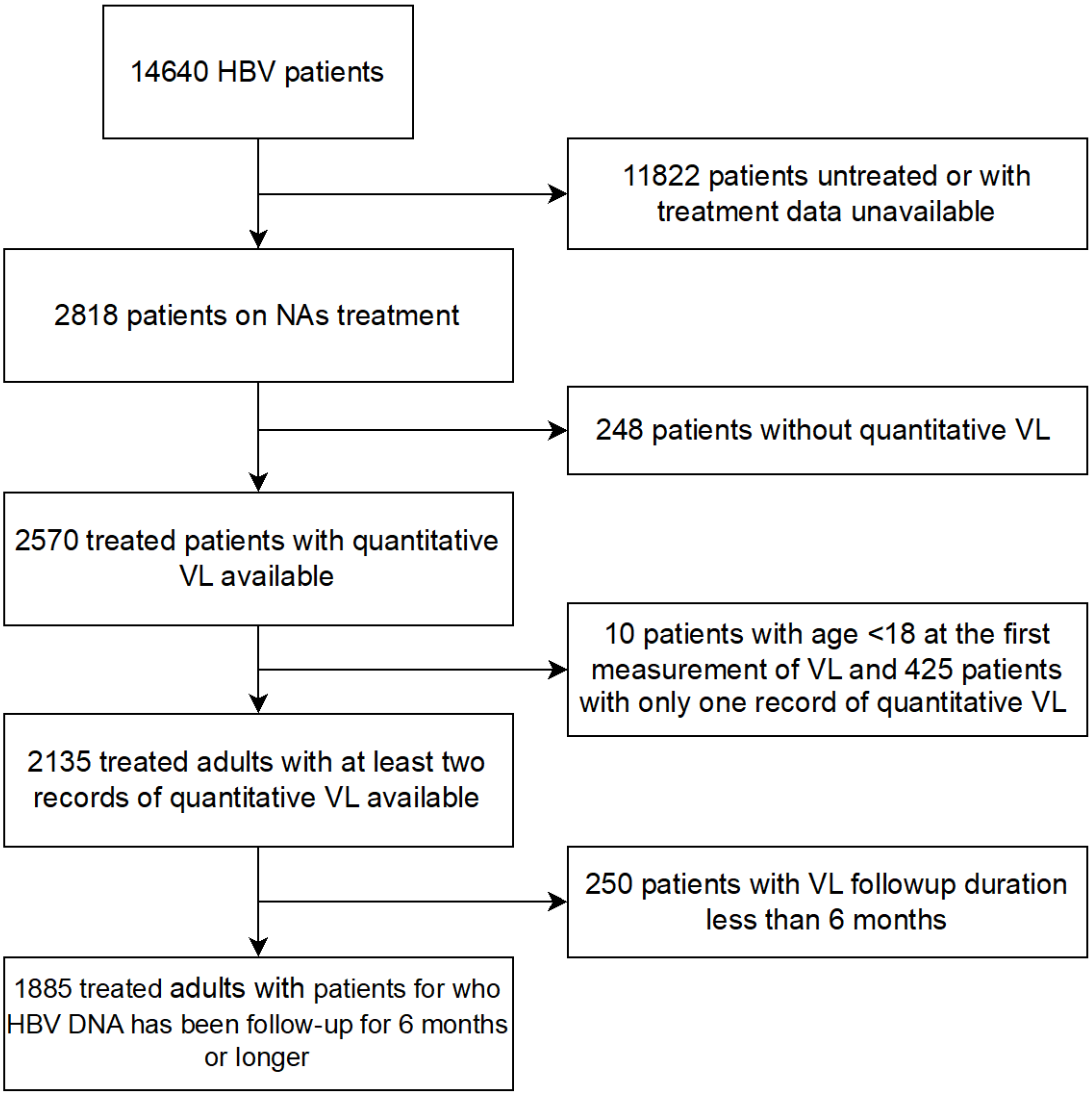

Fig. S1. Flowchart of patient selection for a study of viral load trajectories in adults receiving NA therapy for chronic HBV infection in the UK.

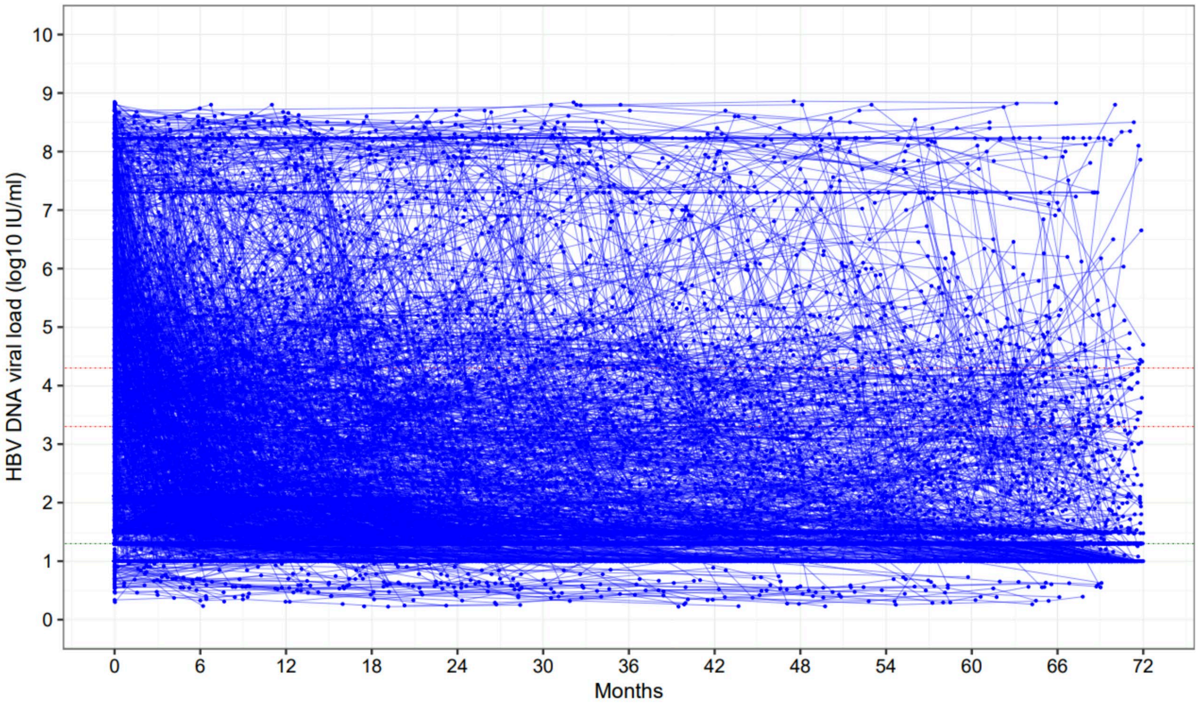

Fig. S2. Overview of individual virologic trajectories in 1885 adults with CHB on NA therapy (prior to the latent class analysis). *In the study cohort, 150 individuals had VL reported as 'detectable' without a quantitative result at some point during their clinical record. This typically suggests a level of viraemia that is below the limit of quantification but at which the assay can still detect the presence of HBV DNA. However, in the majority of these (103/150) the unquantified value occurred in the middle of a longitudinal record of quantitative VL measurements and was therefore not likely to influence the class assigned by the model. To avoid assigning an arbitrary numerical value to qualitative results, we therefore excluded VL datapoints that had a qualitative-only result.*

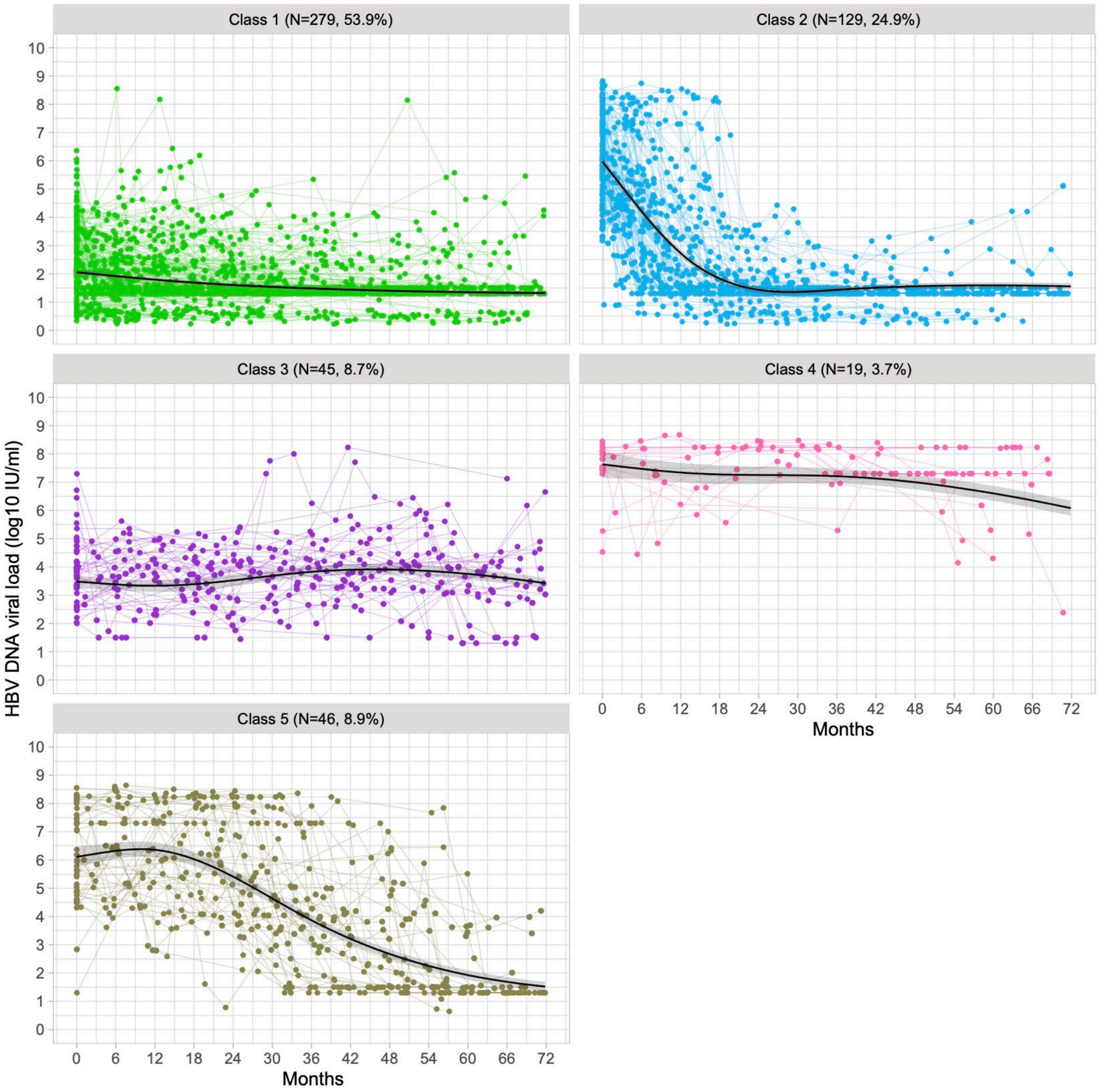

Fig. S3. Individual trajectories of HBV DNA viral load (VL) against the identified five VL trajectory patterns ('classes 1-5') for chronic hepatitis B patients on treatment in validation cohort (n=518). *Individual VL trajectories of validation cohort were classified using the estimated model based on derivation cohort. Dots represent the real values of VL, and solid lines with shading area represent the predicted VL trajectory patterns with 95% confidence intervals.*

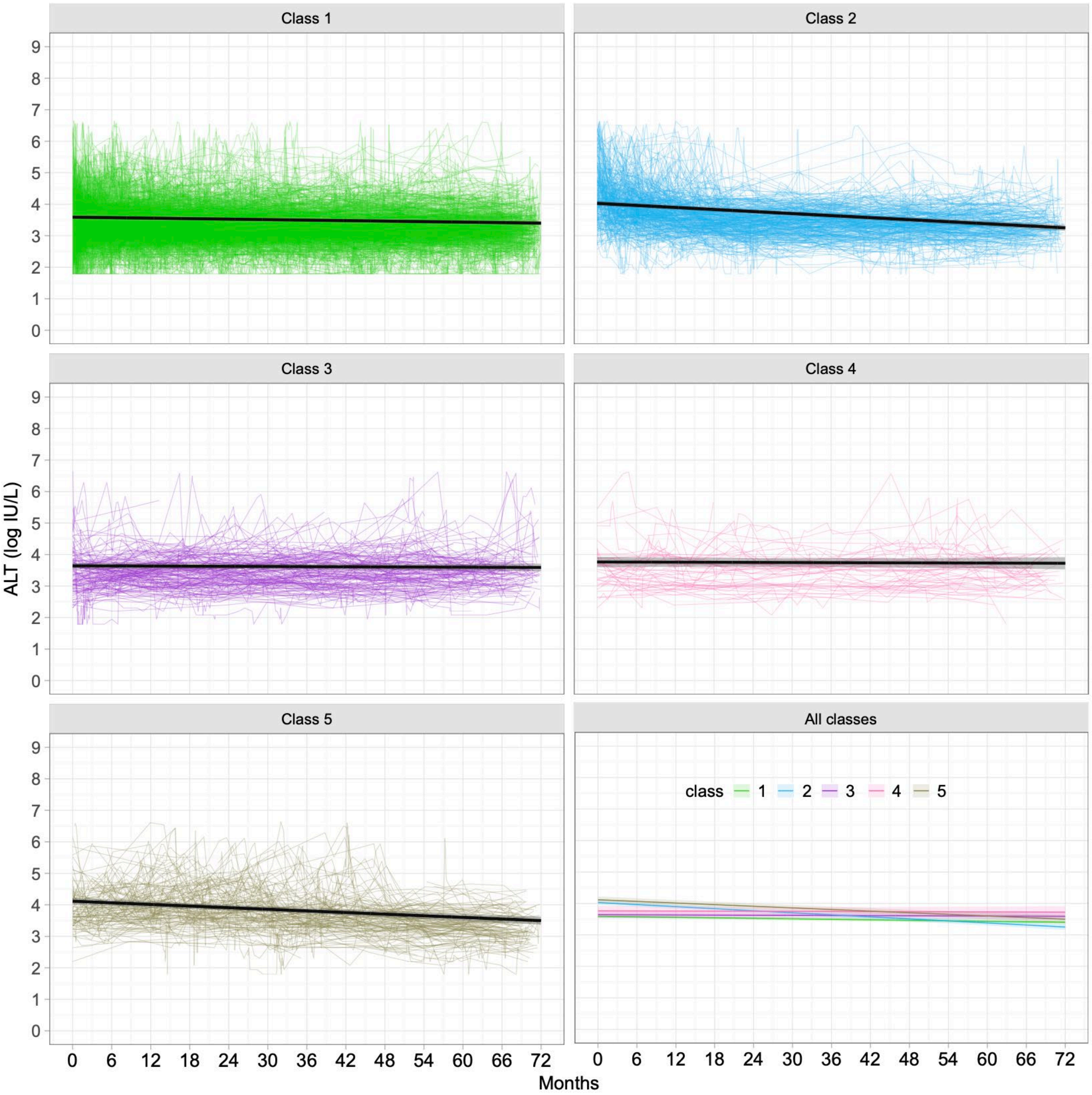

Fig. S4. Longitudinal trend of ALT levels (log IU/L) stratified by VL trajectory patterns in overall study cohort (n=1883, combining derivation and validation cohorts according to their identified VL trajectories, ALT data unavailable for three patients). *The trend was assessed by linear mixed effects models, considering the repeated measurements of each individual patient* and adjusted for age, sex, and ethnicity. *Coloured bands of the solid lines in last panel represent 95% confidence intervals.*

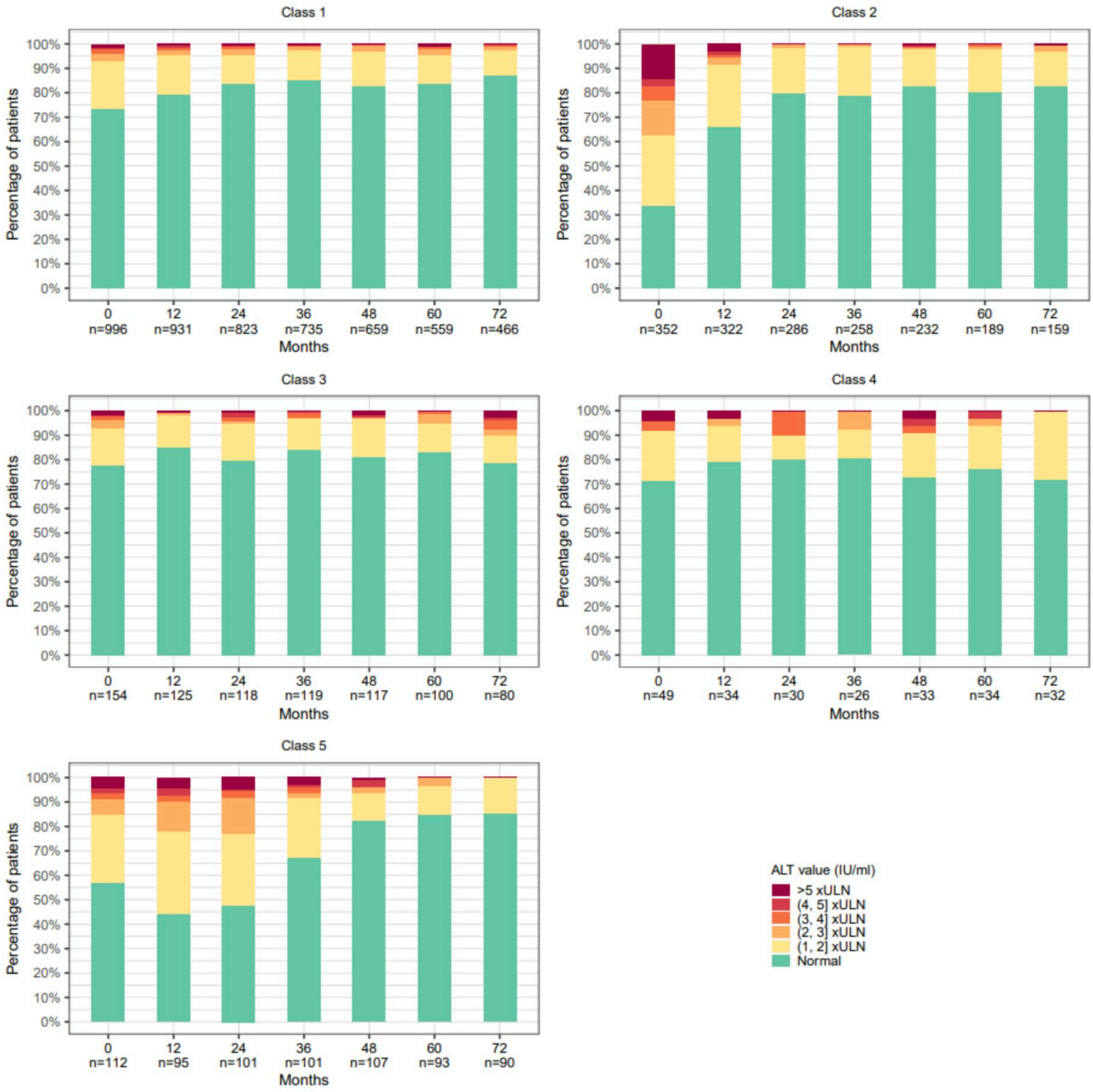

Fig. S5. Stratification of ALT levels over time for patients with distinct virologic trajectory patterns. *ALT, Alanine aminotransferase. ULN, upper limit of normal.*

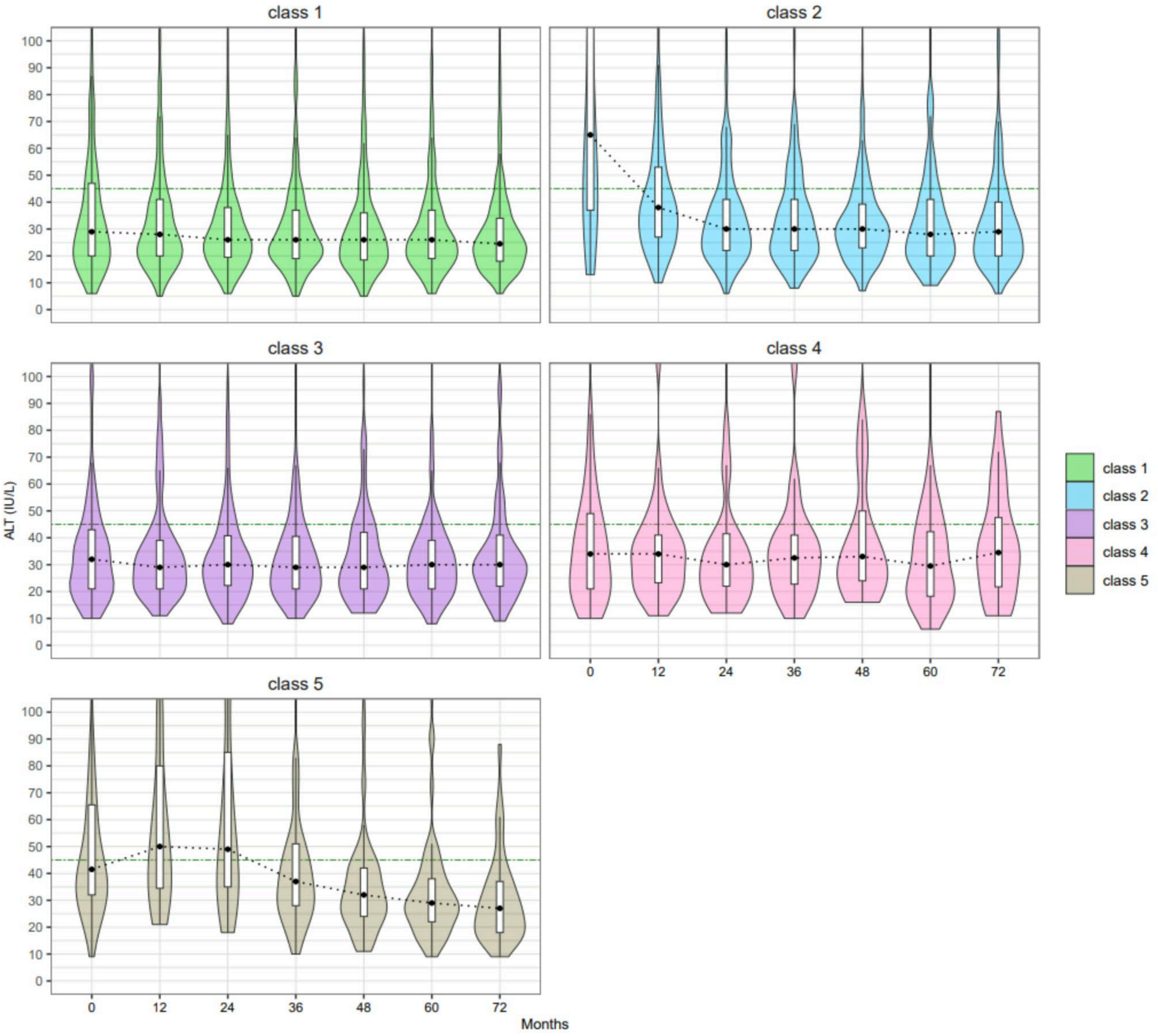

Fig. S6. Distribution of ALT levels at each time point for patients with distinct virologic trajectory patterns. *Green dash-dotted lines indicate the upper limit of normal (ULN) of ALT. Black dotted lines indicate the median values of ALT at each time point. The box represents the median and interquartile range, and the whiskers represent the adjacent values, which are within 1.5 times the interquartile range.*

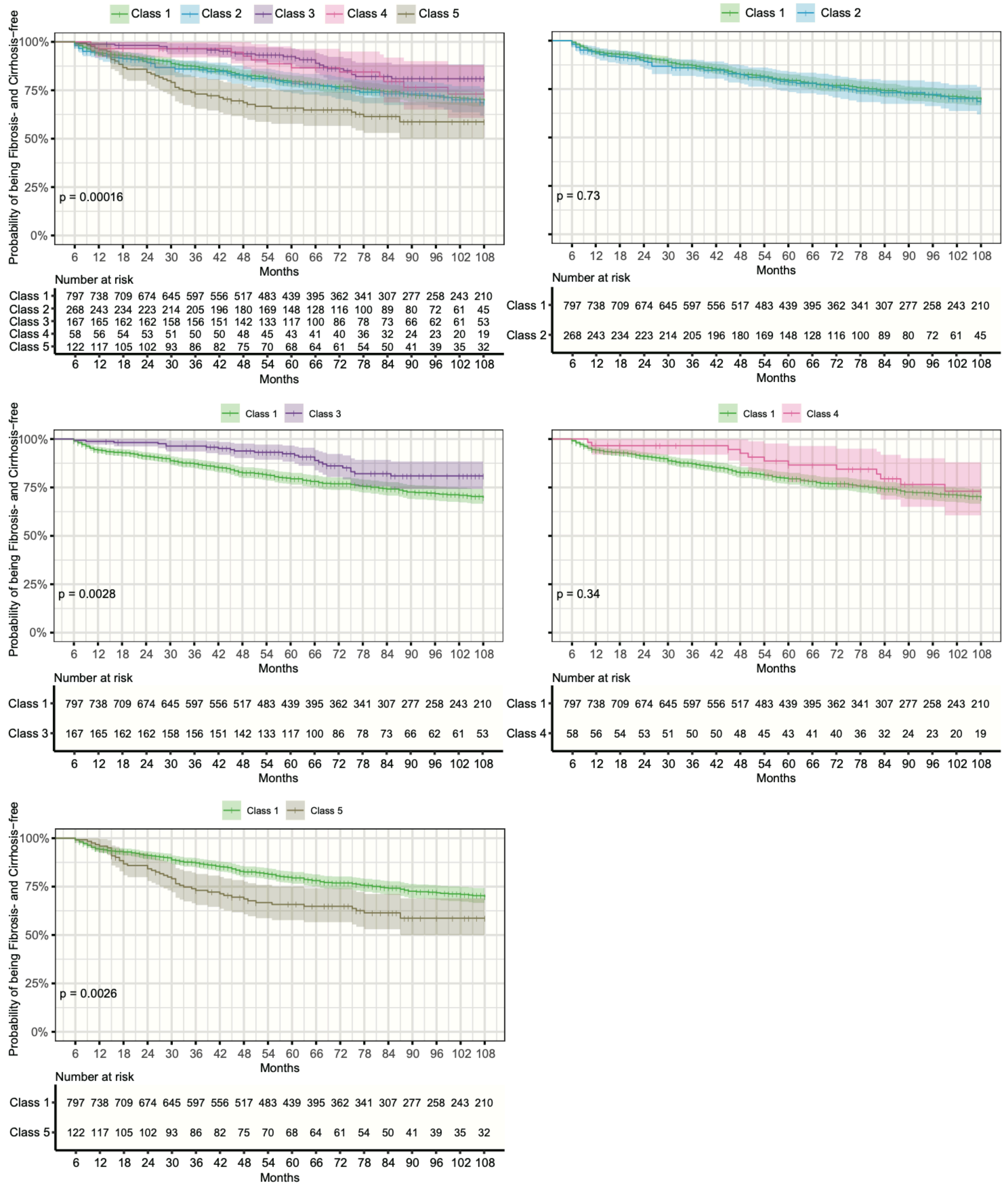

Fig. S7. Progression to liver fibrosis and/or cirrhosis and VL trajectories of adult population with chronic HBV infection treated with NA therapy plotted as Kaplan-Meier (K-M) curves. *K-M curves were truncated at 75% quantiles of follow up duration (months=108) of liver fibrosis/cirrhosis status. Shading areas represent 95% confidence intervals. The shape "|" indicates censoring. Note that classes 3 and 4 are characterised by being more female and younger (see Figure 2), which may be associated with slower rate of progression. HBV, Hepatitis B Virus; VL, viral load; NA, nucleos/tide analogue.*

Table S1. Laboratory parameters used in this study

Category	Laboratory parameter	Full name
HBV lab tests	HBsAg	Hepatitis B surface antigen
	HBV DNA VL	HBV DNA viral load
	HBeAg	Hepatitis B e-Antigen
	Anti-HBe	Anti-Hepatitis B e-antigen
Liver biochemistry and other tests reflecting liver health	ALT	Alanine aminotransferase
	AST	Aspartate aminotransferase
	Albumin	
	ALP	Alkaline phosphatase
	Bilirubin	
	Platelets	
Renal function	eGFR	Estimated Glomerular Filtration Rate
	Urea	
Virology tests for other chronic viral coinfections inference	HIV	Human immunodeficiency virus
	HCV	Hepatitis C virus
	HDV	Hepatitis D virus

Table S2. Calculation of discrimination and entropy

<table>
<tr><th>Metrics</th><th>Formula</th><th>Note</th></tr>
<tr><td>Discrimination</td><td>$\frac{\sum_{i=1}^{N} \max\{P_i^k, k = 1, 2, 3, K\}}{N}$</td><td rowspan="2">$K$ represents the number of classes, N indicates the number of patients included in the cohort for modelling. P_i^k indicates the probability of patient i being assigned to class k.</td></tr>
<tr><td>Entropy</td><td>$1 + \sum_{i=1}^{N} \frac{\sum_k^K \log(P_i^k) \cdot P_i^k}{N \cdot \log(K)}$</td></tr>
</table>

Table S3. Performance of the models with different number of classes of VL trajectories in the derivation cohort.

Number of classes	BIC	SABIC	AIC	Entropy	Proportion for each class
1	55654	55629	55612	1	100%
2	54017	53954	53913	0.86	78.3%: 21.7%
3	53212	53110	53045	0.88	72.7%: 19.6%: 7.7%
4	52361	52221	52131	0.91	66.9%: 18.4%: 7.0%: 7.7%
5	52124	51946	51832	0.90	60.5%: 18.6%: 10.2%: 3.2%: 7.5%
6	51868	51652	51513	0.90	59.6%: 18.5%: 3.3%: 9.1%: 3.1%: 6.3%

Table S4. Classification performance of final estimated model in the derivation and validations cohorts

**Assessment metrics**	**Derivation cohort**	**Validation cohort**
Discrimination	0.93	0.9287
Entropy	0.90	0.8815
APPA for each class	0.9509: 0.9221: 0.8543: 0.9436: 0.9330	0.9370: 0.9224: 0.8643: 0.9751: 0.9403
Proportions per class	60.5%: 18.6%: 10.2%: 3.2%: 7.5%	53.9%: 24.9%: 8.7%: 3.7%: 8.9%

APPA, average posterior probability assignment

Table S5. Characteristics of patients at presentation stratified by the virologic trajectory patterns identified by latent class mixed modelling in the derivation cohort (n=1367)

**Characteristics**	**Class 1**<br>**N=827 (60.5%)**	**Class 2**<br>**N=254 (18.6%)**	**Class 3**<br>**N=140 (10.2%)**	**Class 4**<br>**N=44 (3.2%)**	**Class 5**<br>**N=102 (7.5%)**	***p* value**
	Long term suppression	Timely virological suppression	Persistent moderate viraemia	Persistent high-level viraemia	Slow virological suppression	
Sex, male	526 (63.6)	66 (47.1)	526 (63.6)	179 (70.5)	9 (20.5)	**<0.001**
Age, years	47 [37, 58]	44 [34, 55]	38 [33, 45]	30 [23, 35]	36 [28, 46]	**<0.001**
Age group, years						**<0.001**
18-24	16 (1.9)	7 (2.8)	4 (2.9)	14 (31.8)	14 (13.7)	
25-34	149 (18.0)	62 (24.4)	43 (30.7)	18 (40.9)	32 (31.4)	
35-44	187 (22.6)	59 (23.2)	57 (40.7)	10 (22.7)	25 (24.5)	
45-54	205 (24.8)	59 (23.2)	30 (21.4)	2 (4.5)	19 (18.6)	
55-64	147 (17.8)	43 (16.9)	5 (3.6)	0 (0.0)	11 (10.8)	
65-74	94 (11.4)	17 (6.7)	1 (0.7)	0 (0.0)	1 (1.0)	
>=75	29 (3.5)	7 (2.8)	0 (0.0)	0 (0.0)	0 (0.0)	
Ethnic group						
Asian	211 (30.8)	86 (41.7)	62 (47.7)	23 (67.6)	36 (43.4)	**<0.001**
Black	175 (25.5)	34 (16.5)	19 (14.6)	6 (17.6)	9 (10.8)	
White	172 (25.1)	44 (21.4)	18 (13.8)	1 (2.9)	25 (30.1)	
Mixed/other ethnicity	127 (18.5)	42 (20.4)	31 (23.8)	4 (11.8)	13 (15.7)	
Not reported	142 (17.2)	48 (18.9)	10 (7.1)	10 (22.7)	19 (18.6)	
IMD (decile)	4.0 [2.0, 6.0]	4.0 [2.0, 6.0]	4.0 [2.0, 7.0]	3.0 [1.0, 6.0]	3.0 [1.8, 6.0]	**0.015**
IMD not reported	66 (8.0)	23 (9.1)	52 (37.1)	5 (11.4)	10 (9.8)	**<0.001**

HBeAg status						
Negative	323 (80.8)	98 (53.8)	90 (89.1)	3 (7.5)	27 (32.5)	**<0.001**
Positive	77 (19.2)	84 (46.2)	11 (10.9)	37 (92.5)	56 (67.5)	
Not available	427 (51.6)	72 (28.3)	39 (27.9)	4 (9.1)	19 (18.6)	
Anti-HBe status						
Negative	125 (26.2)	74 (41.1)	15 (14.6)	37 (92.5)	54 (65.9)	**<0.001**
Positive	353 (73.8)	106 (58.9)	88 (85.4)	3 (7.5)	28 (34.1)	
Not available	349 (42.2)	74 (29.1)	37 (26.4)	4 (9.1)	20 (19.6)	
HBV VL, log10 IU/ml	1.5 [1.3, 2.5]	6.3 [5.1, 7.7]	3.6 [2.8, 4.5]	8.2 [7.5, 8.5]	6.3 [4.4, 8.2]	**<0.001**
HBV VL category, IU/ml						**<0.001**
<20	118 (14.3)	0 (0.0)	1 (0.7)	0 (0.0)	1 (1.0)	
20 - <2000	590 (71.3)	3 (1.2)	52 (37.1)	2 (4.5)	6 (5.9)	
2000 - <20,000	77 (9.3)	15 (5.9)	50 (35.7)	0 (0.0)	13 (12.7)	
>=20,000	42 (5.1)	236 (92.9)	37 (26.4)	42 (95.5)	82 (80.4)	
ALT, IU/L	28 [19, 45]	70 [38, 147]	31 [21, 40]	35 [25, 50]	42 [33, 73]	**<0.001**
AST, IU/L	30 [24, 40]	48 [34, 79]	27 [23, 32]	27 [22, 51]	32 [28, 54]	**<0.001**
Platelets, 10^9/L	199 [158, 248]	193 [155, 231]	218 [169, 257]	203 [177, 247]	221 [178, 270]	**0.001**
Albumin, g/L	40.0 [36.0, 43.0]	40.0 [37.0, 42.0]	43.0 [39.2, 45.8]	39.0 [35.8, 43.2]	41.0 [38.0, 43.0]	**<0.001**
ALP, IU/L	79.0 [62.0, 98.0]	80.0 [61.0, 103.5]	64.0 [52.0, 80.8]	64.5 [60.0, 78.2]	70.5 [61.0, 88.2]	**<0.001**
Bilirubin, µmol/L	9.0 [6.0, 13.0]	11.0 [7.5, 15.0]	8.5 [6.0, 12.0]	10.0 [6.0, 11.5]	10.0 [7.5, 13.0]	**0.001**
eGFR category, mL/min/1.73 m^2						**<0.001**
>=90	251 (40.2)	75 (43.1)	53 (54.1)	9 (75.0)	37 (64.9)	
>=60 & <=89	265 (42.5)	88 (50.6)	38 (38.8)	3 (25.0)	16 (28.1)	

<=59	108 (17.3)	11 (6.3)	7 (7.1)	0 (0.0)	4 (7.0)	
Not available	203 (24.5)	80 (31.5)	42 (30.0)	32 (72.7)	45 (44.1)	
Urea, mmol/L	5.0 [4.0, 6.5]	4.8 [3.9, 5.8]	4.8 [3.9, 5.9]	3.6 [3.2, 4.4]	5.1 [4.1, 6.0]	**<0.001**
Urea, not available	39 (4.7)	16 (6.3)	27 (19.3)	12 (27.3)	25 (24.5)	
Treatment regimens						**<0.001**
TDF	468 (56.6)	189 (74.4)	94 (67.1)	33 (75.0)	63 (61.8)	
ETV	161 (19.5)	30 (11.8)	25 (17.9)	3 (6.8)	12 (11.8)	
ETV+TDF	39 (4.7)	19 (7.5)	9 (6.4)	3 (6.8)	12 (11.8)	
LAM/ADE+TDF	30 (3.6)	6 (2.4)	2 (1.4)	1 (2.3)	6 (5.9)	
LAM/ADE+ETV	14 (1.7)	0 (0.0)	2 (1.4)	0 (0.0)	1 (1.0)	
Other regimens†	115 (13.9)	10 (3.9)	8 (5.7)	4 (9.1)	8 (7.8)	

Data are the number (%) or median [IQR]. IMD, Index of Multiple Deprivation; VL, viral load; ALT, alanine aminotransferase; TDF, tenofovir disoproxil fumarate; ETV, entecavir; LAM, lamivudine; ADE, adefovir. Comparison was conducted across non-missing categories for a categorical variable. † Four patients were on interferon only, 113 patients were on single drug of LAM or ADE, 18 patients were on interferon combined with TDF and/or ETV, 10 patients on a combination of LAM, TDF, and ETV.

Table S6. Characteristics of patients at presentation stratified by the virologic trajectory patterns identified by latent class mixed modelling in the validation cohort (n=518).

**Characteristics**	**Class 1**<br>**N =279 (53.9%)**	**Class 2**<br>**N=129 (24.9%)**	**Class 3**<br>**N=45 (8.7%)**	**Class 4**<br>**N=19 (3.7%)**	**Class 5**<br>**N=46 (8.9%)**
	Long term suppression	Timely virological suppression	Persistent moderate viraemia	Persistent high-level viraemia	Slow virological suppression
Sex, male	189 (67.7)	87 (67.4)	26 (57.8)	11 (57.9)	25 (54.3)
Age, years	43 [35, 55]	39 [32, 48]	39 [33, 46]	31 [24, 38]	36 [31, 48]
Age group, years					
18-24	3 (1.1)	9 (7.0)	1 (2.2)	5 (26.3)	4 (8.7)
25-34	64 (22.9)	37 (28.7)	16 (35.6)	7 (36.8)	14 (30.4)
35-44	87 (31.2)	39 (30.2)	14 (31.1)	4 (21.1)	16 (34.8)
45-54	54 (19.4)	25 (19.4)	9 (20.0)	1 (5.3)	5 (10.9)
55-64	49 (17.6)	15 (11.6)	5 (11.1)	2 (10.5)	7 (15.2)
65-74	17 (6.1)	1 (0.8)	0 (0.0)	0 (0.0)	0 (0.0)
>=75	5 (1.8)	3 (2.3)	0 (0.0)	0 (0.0)	0 (0.0)
Ethnic group					
Asian	107 (44.6)	46 (44.2)	22 (51.2)	9 (56.2)	22 (52.4)
Black	44 (18.3)	16 (15.4)	6 (14.0)	2 (12.5)	3 (7.1)
White	73 (30.4)	33 (31.7)	12 (27.9)	3 (18.8)	12 (28.6)
Mixed/other ethnicity	16 (6.7)	9 (8.7)	3 (7.0)	2 (12.5)	5 (11.9)
Not reported	39 (14.0)	25 (19.4)	2 (4.4)	3 (15.8)	4 (8.7)

IMD (decile)	6.0 [4.0, 9.0]	7.0 [5.0, 9.0]	7.0 [5.0, 9.0]	6.0 [5.0, 8.0]	6.0 [4.0, 9.0]
IMD category					
20% most deprived	28 (10.6)	12 (9.8)	2 (4.7)	1 (5.6)	5 (11.4)
20% to 40%	49 (18.6)	18 (14.8)	7 (16.3)	3 (16.7)	8 (18.2)
40% to 60%	57 (21.7)	26 (21.3)	12 (27.9)	8 (44.4)	11 (25.0)
60% to 80%	61 (23.2)	31 (25.4)	9 (20.9)	2 (11.1)	7 (15.9)
20% least deprived	68 (25.9)	35 (28.7)	13 (30.2)	4 (22.2)	13 (29.5)
Not reported	16 (5.7)	7 (5.4)	2 (4.4)	1 (5.3)	2 (4.3)
HBeAg status					
Negative	169 (80.1)	60 (48.8)	26 (78.8)	0 (0.0)	18 (47.4)
Positive	42 (19.9)	63 (51.2)	7 (21.2)	17 (100.0)	20 (52.6)
Not available	68 (24.4)	6 (4.7)	12 (26.7)	2 (10.5)	8 (17.4)
Anti-HBe status					
Negative	51 (25.0)	64 (52.0)	6 (18.2)	16 (94.1)	18 (50.0)
Positive	153 (75.0)	59 (48.0)	27 (81.8)	1 (5.9)	18 (50.0)
Not available	75 (26.9)	6 (4.7)	12 (26.7)	2 (10.5)	10 (21.7)
HBV VL, log10 IU/ml	2.1 [1.4, 3.4]	6.4 [5.0, 7.7]	3.9 [3.2, 4.6]	8.0 [7.5, 8.2]	7.1 [5.1, 8.1]
HBV VL category, IU/ml					
<20	46 (16.5)	0 (0.0)	0 (0.0)	0 (0.0)	0 (0.0)
20 - <2000	173 (62.0)	1 (0.8)	13 (28.9)	0 (0.0)	2 (4.3)

2000 - <20,000	44 (15.8)	14 (10.9)	18 (40.0)	0 (0.0)	0 (0.0)
>=20,000	16 (5.7)	114 (88.4)	14 (31.1)	19 (100.0)	44 (95.7)
ALT, IU/L	33.0 [23.0, 51.0]	58.0 [34.0, 102.0]	34.5 [22.0, 44.5]	34.0 [17.5, 49.5]	39.5 [30.2, 62.8]
AST, IU/L	35.5 [27.0, 46.8]	42.5 [34.5, 66.5]	26.5 [20.5, 36.5]	15.0 [15.0, 15.0]	35.0 [30.0, 56.0]
Platelets, 10^9/L	185.0 [150.0, 232.0]	206.0 [168.0, 239.0]	217.0 [174.0, 230.0]	203.0 [193.0, 239.0]	199.5 [179.8, 232.0]
Albumin, g/L	40.0 [38.0, 43.0]	40.0 [35.0, 42.0]	41.0 [40.0, 44.0]	36.0 [31.0, 40.0]	39.0 [35.0, 41.2]
ALP, IU/L	74.0 [61.0, 96.0]	84.0 [65.0, 108.0]	67.5 [59.5, 79.8]	87.0 [71.0, 107.0]	71.5 [59.5, 88.5]
Bilirubin, μmol/L	11.0 [8.0, 14.0]	11.0 [8.0, 15.0]	10.0 [6.8, 16.5]	7.0 [5.0, 9.0]	9.5 [6.8, 12.2]
eGFR category, mL/min/1.73 m^2					
>=90	146 (59.3)	72 (64.9)	13 (41.9)	8 (80.0)	15 (44.1)
>=60 & <=89	85 (34.6)	37 (33.3)	16 (51.6)	2 (20.0)	19 (55.9)
<=59	15 (6.1)	2 (1.8)	2 (6.5)	0 (0.0)	0 (0.0)
Not available	33 (11.8)	18 (14.0)	14 (31.1)	9 (47.4)	12 (26.1)
Urea, mmol/L	5.0 [4.2, 6.1]	4.9 [4.0, 5.8]	4.6 [3.9, 5.0]	4.2 [3.9, 4.8]	4.9 [4.2, 5.8]
Treatment regimens					
TDF	94 (33.7)	64 (49.6)	21 (46.7)	7 (36.8)	15 (32.6)
ETV	73 (26.2)	38 (29.5)	15 (33.3)	5 (26.3)	17 (37.0)
ETV+TDF	25 (9.0)	19 (14.7)	8 (17.8)	6 (31.6)	10 (21.7)
LAM/ADE+TDF	28 (10.0)	2 (1.6)	1 (2.2)	0 (0.0)	0 (0.0)
LAM/ADE+ETV	33 (11.8)	1 (0.8)	0 (0.0)	0 (0.0)	0 (0.0)
Other regimens	26 (9.3)	5 (3.9)	0 (0.0)	1 (5.3)	4 (8.7)

Data are the number (%) or median [IQR]. IMD, Index of Multiple Deprivation; VL, viral load; ALT, alanine aminotransferase; TDF, tenofovir disoproxil fumarate; ETV, entecavir; LAM, lamivudine; ADE, adefovir. Significance tests were not conducted in the validation cohort due to small numbers in some classes.

Table S7. Data source for determining liver fibrosis or cirrhosis

**Data source**	**Number of patients with evidence of liver fibrosis or cirrhosis by each source**
Biopsy	43 (13%)
Elastography (FibroScan)	34 (10%)
Imaging (Ultrasound/CT/MRI)	22 (7%)
ICD	78 (23%)
FIB4 or APRI	155 (47%)
Total	332

CT – computer tomography / MRI – magnetic resonance imaging

ICD – international classification of disease

FIB4 and APRI – laboratory based liver fibrosis scores

Table S8. Comparisons of probability of being liver fibrosis and cirrhosis-free status over time for classes with distinct VL trajectory patterns

	Class 1	Class 2	Class 3	Class 4
Class 2	0.727	**-**	**-**	**-**
Class 3	**0.003**	**0.009**	**-**	
Class 4	0.340	0.397	0.466	**-**
Class 5	**0.003**	**0.034**	**<0.0001**	**0.029**

Data were p values. The pairwise comparisons were based on log-rank test.

Table S9. Univariate and multivariate Cox proportional-hazards models investigating associations of different VL trajectory patterns with liver disease progression to fibrosis and/or cirrhosis among adults with chronic HBV infection on NA therapy

	**Univariate analysis**		**Multivariate analysis**	
**Variables**	**Crude HR (95% CIs)**	***p* value**	**Adjusted HR (95% CI)**	***p* value**
VL trajectory				
Class 1 (reference)	1.0		1.0	
Class 2	1.05 (0.79 - 1.40)	0.734	1.08 (0.77 - 1.51)	0.654
Class 3	0.53 (0.35 - 0.81)	**0.004**	0.78 (0.50 - 1.23)	0.286
Class 4	0.75 (0.42 - 1.35)	0.338	1.55 (0.79 - 3.01)	0.199
Class 5	1.63 (1.18 - 2.25)	**0.003**	2.24 (1.55 - 3.24)	**<0.001**
Age group				
18-24 years	1.54 (0.87 - 2.72)	0.137	1.64 (0.9 - 3.01)	0.109
25-34 years (reference)	1.0		1.0	
35-44 years	1.12 (0.8 - 1.58)	0.515	1.13 (0.79 - 1.6)	0.499
45-54 years	1.52 (1.08 - 2.14)	**0.016**	1.31 (0.91 - 1.9)	0.150
55-64 years	2.34 (1.65 - 3.33)	**<0.001**	1.95 (1.33 - 2.85)	**<0.001**
65-74 years	3.36 (2.16 - 5.23)	**<0.001**	3.13 (1.93 - 5.07)	**<0.001**
>=75 years	5.24 (2.74 - 10.02)	**<0.001**	5.70 (2.84 - 11.44)	**<0.001**
Sex				
Female (reference)	1.0		1.0	
Male	1.59 (1.25 - 2.02)	**<0.001**	1.40 (1.06 - 1.84)	**0.016**
Ethnic group				
White (reference)	1.0		1.0	
Asian	0.81 (0.61 - 1.09)	0.163	0.91 (0.68 - 1.22)	0.522
Black	1.17 (0.85 - 1.62)	0.338	1.29 (0.92 - 1.82)	0.141
Mixed or other ethnicity	1.02 (0.72 - 1.46)	0.896	1.07 (0.74 - 1.55)	0.732
IMD				
20% most deprived	1.01 (0.72 - 1.41)	0.966	0.94 (0.67 - 1.31)	0.719
20% to 40%	1.14 (0.81 - 1.59)	0.445	1.21 (0.86 - 1.70)	0.275
40% to 60% (reference)	1.0		1.0	
60% to 80%	1.21 (0.83 - 1.77)	0.320	1.23 (0.86 - 1.78)	0.262
20% least deprived	1.23 (0.83 - 1.81)	0.305	1.27 (0.86 - 1.90)	0.231
Coinfection† (Yes)	1.6 (1.01 - 2.51)	**0.043**	1.09 (0.65 - 1.84)	0.748
HBeAg status (Positive)	1.01 (0.77 - 1.32)	0.939	1.01 (0.60 - 1.68)	0.982
Anti-HBe status (Positive)	1.01 (0.79 - 1.29)	0.928	1.52 (0.94 - 2.46)	0.091
Albumin, g/L	0.94 (0.93, 0.96)	**<0.001**	0.95 (0.93 - 0.97)	**<0.001**

ALP (divided by 10), IU/L	1.03 (1.02, 1.04)	**<0.001**	1.03 (1.01 - 1.04)	**<0.001**
AST (divided by 10), IU/L	1.07 (1.05, 1.10)	**<0.001**	1.07 (1.04 - 1.10)	**<0.001**
ALT (divided by 10), IU/L	1.01 (0.99, 1.01)	0.199	0.99 (0.98 - 1.01)	0.239
Bilirubin (divided by 10), μmol/L	1.03 (0.99, 1.07)	0.074	1.02 (0.97 - 1.08)	0.422
Platelets (divided by 10), 10^9/L	0.94 (0.92, 0.96)	**<0.001**	0.94 (0.92 - 0.96)	**<0.001**
Haemoglobin (divided by 10), g/L	1.01 (0.99, 1.03)	0.113	1.01 (0.99 - 1.03)	0.308
HBV VL‡, log10 IU/ml	0.98 (0.93, 1.03)	0.40		
eGFR category				
≥90 mL/minute/1.73 m^2 (reference)	1.0		1.0	
≥60 and ≤89 mL/minute/1.73 m^2	1.09 (0.85 - 1.39)	0.500	0.88 (0.68 - 1.13)	0.303
≤59 mL/minute/1.73 m^2	1.87 (1.36 - 2.58)	**<0.001**	0.95 (0.60 - 1.51)	0.834
Urea, mmol/L	1.06 (1.03 - 1.08)	**<0.001**	1.00 (0.96 - 1.04)	0.938
Treatment regimens				
TDF (reference)	1.0		1.0	
ETV	1.19 (0.89 - 1.58)	0.239	0.92 (0.68 - 1.25)	0.601
ETV+TDF	1.86 (1.32 - 2.62)	**<0.001**	1.66 (1.16 - 2.37)	**0.006**
LAM/ADE+TDF	1.62 (0.97 - 2.71)	0.066	1.14 (0.65 - 1.98)	0.655
LAM/ADE+ETV	2.21 (1.20 - 4.09)	**0.011**	1.32 (0.69 - 2.55)	0.404
Other regimens	1.56 (1.06 - 2.28)	**0.022**	1.06 (0.69 - 1.62)	0.791

† Coinfection with HIV, HCV, or HDV.

‡ The variable of HBV VL levels at baseline was not separately accounted for the multivariate analysis because the VL trajectory already incorporates the HBV VL levels at baseline. 1412 patients with data available for identifying liver fibrosis and cirrhosis were included for multivariate analysis. Class 1 (VL long term suppressed), Class 2 (persistent viraemia with moderate VL), Class 3 (VL suppressed as expected), Class 4 (VL non-suppressing with high VL), Class 5 (VL slowly suppressed). VL, viral load; ALT, Alanine transaminase; ALP, Alkaline phosphatase; HR, Hazards ratio, TDF, tenofovir disoproxil fumarate; ETV, entecavir; LAM, lamivudine; ADE, adefovir.

Table S10. Sensitivity analysis only adjusting for age and sex for investigating associations of different VL trajectory patterns with liver disease progression to fibrosis and/or cirrhosis among adults with chronic HBV infection on NA therapy

**Variables**	**Adjusted HR (95% CI)**	***p* value**
VL trajectory		
Class 1 (reference)	1.0	
Class 2	1.15 (0.86 – 1.53)	0.354
Class 3	0.72 (0.47 – 1.11)	0.138
Class 4	1.15 (0.62 – 2.13)	0.665
Class 5	1.93 (1.38 – 2.69)	**<0.001**
Age group		
18-24 years	1.40 (0.78 – 2.51)	0.264
25-34 years (reference)	1.0	
35-44 years	1.10 (0.78 – 1.55)	0.583
45-54 years	1.50 (1.06 – 2.13)	**0.021**
55-64 years	2.20 (1.54 – 3.15)	**<0.001**
65-74 years	3.28 (2.09 – 5.15)	**<0.001**
>=75 years	5.70 (2.95 – 11.01)	**<0.001**
Sex		
Female (reference)	1.0	
Male	1.52 (1.19 – 1.94)	**<0.001**

Table S11. Predictive ability (AUC, Sensitivity, Specificity) of each single variable (VL trajectory, demographics, and biochemistry parameters) for liver fibrosis and cirrhosis

Variables	AUC	Sensitivity	Specificity	Accuracy	p-value (compared to VL trajectory)
VL trajectory	0.658 (0.624-0.691)	0.593	0.650	0.637	-
Baseline age	0.666 (0.632-0.699)	0.581	0.655	0.637	0.62
Sex	0.635 (0.601-0.670)	0.554	0.604	0.592	0.10
Platelets	0.678 (0.643-0.711)	0.614	0.645	0.638	0.26
AST	0.642 (0.608-0.676)	0.542	0.678	0.646	0.20
Albumin	0.655 (0.622-0.689)	0.569	0.649	0.630	0.87
ALP	0.655 (0.621-0.689)	0.566	0.676	0.650	0.83
Treatment regimens	0.653 (0.619-0.687)	0.578	0.670	0.649	0.75

Note: ALP, Alkaline phosphatase; ALT, Alanine transaminase; VL, viral load; ROC, receiver operating characteristic; AUC, the area under an ROC curve.

Table S12. Improvement of predictive ability of the addition of VL trajectories, other biochemistry parameters, and treatment regimens for liver fibrosis and cirrhosis

Predictors	AUC	Sensitivity	Specificity	Accuracy	p-value (compared to preceding row)
Baseline age + sex	0.669 (0.635, 0.703)	0.590	0.636	0.625	-
Baseline age + sex + PLT + AST + ALB + ALP	0.731 (0.699, 0.763)	0.639	0.688	0.676	**<0.001**
Baseline age + sex + PLT + AST + ALB + ALP + Treatment regimens	0.738 (0.706, 0.770)	0.645	0.700	0.687	0.14
Baseline age + sex + PLT + AST + ALB + ALP + Treatment regimens + VL trajectory	0.756 (0.725, 0.787)	0.684	0.714	0.707	**<0.01**

All the models adjusted for other parameters that were included in the multivariate analysis, including ethnic group, IMD, HBeAg, anti-HBe, coinfection, ALT, Bilirubin, Hb, eGFR, and Urea.

Table S13. Sensitivity analysis excluding NA agents other than tenofovir and entecavir for investigating associations of different VL trajectory patterns with liver disease progression to fibrosis and/or cirrhosis among adults with chronic hepatitis B on NA therapy

**Variables**	**Adjusted HR (95% CI)**	***p* value**
VL trajectory		
Class 1 (reference)	1.0	
Class 2	1.21 (0.86 - 1.71)	0.267
Class 3	0.85 (0.53 - 1.37)	0.507
Class 4	1.57 (0.79 - 3.14)	0.199
Class 5	2.54 (1.73 - 3.71)	**<0.001**
Age group		
18-24 years	1.51 (0.81 - 2.81)	0.196
25-34 years (reference)	1.0	
35-44 years	1.03 (0.71 - 1.49)	0.897
45-54 years	1.23 (0.82 - 1.83)	0.318
55-64 years	1.54 (1.01 - 2.34)	**0.043**
65-74 years	2.68 (1.58 - 4.55)	**<0.001**
>=75 years	4.43 (2.04 - 9.61)	**<0.001**
Sex		
Female (reference)	1.0	
Male	1.31 (0.98 - 1.76)	0.072
Ethnic group		
White (reference)	1.0	
Asian	0.92 (0.67 - 1.27)	0.610
Black	1.13 (0.76 - 1.68)	0.532
Mixed or other ethnicity	1.10 (0.74 - 1.64)	0.627
IMD		
20% most deprived	0.86 (0.59 - 1.27)	0.452
20% to 40%	1.28 (0.87 - 1.86)	0.206
40% to 60% (reference)	1.0	
60% to 80%	1.15 (0.76 - 1.75)	0.500
20% least deprived	1.45 (0.95 - 2.23)	0.088
Coinfection (Yes)	1.14 (0.70 - 1.83)	0.598
HBeAg status (Positive)	0.81 (0.46 - 1.44)	0.475
Anti-HBe status (Positive)	1.27 (0.73 - 2.22)	0.396
Albumin, g/L	0.96 (0.94 - 0.99)	**0.021**
ALP (divided by 10), IU/L	1.05 (1.03 - 1.06)	**<0.001**

AST (divided by 10), IU/L	1.13 (1.09 - 1.18)	**<0.001**
ALT (divided by 10), IU/L	0.99 (0.99 - 1.00)	0.114
Bilirubin (divided by 10), μmol/L	1.00 (0.99 - 1.06)	0.961
Platelets (divided by 10), 10^9/L	0.94 (0.92 -0.96)	**<0.001**
Haemoglobin (divided by 10), g/L	1.01 (0.99 - 1.03)	0.368
eGFR category		
≥90 mL/minute/1.73 m^2 (reference)	1.0	
≥60 and ≤89 mL/minute/1.73 m^2	1.03 (0.78 - 1.35)	0.861
≤59 mL/minute/1.73 m^2	1.44 (0.84 - 2.46)	0.180
Urea, mmol/L	0.99 (0.94 - 1.04)	0.601
Treatment regimens		
TDF (reference)	1.0	
ETV	0.92 (0.68 - 1.24)	0.570
ETV+TDF	1.49 (1.03 - 2.14)	**0.034**

Table S14. Longitudinal trend of quantitative HBsAg level over time for distinct virologic trajectory patterns for patients with chronic HBV infection on NA therapy in the subset (N=273) of the study cohort

	Intercept (95% CI), log10 IU/ml	Coefficient β (95% CI)
Class 1 (VL long term suppressed)	3.86 (3.39, 4.33) ***	-0.0084 (-0.0116, -0.0052) ***
Class 2 (VL suppressed timely)	4.45 (3.95, 4.95) ***	-0.0163 (-0.0217, -0.0108) ***
Class 3 (VL persistent with moderate levels)	4.06 (3.57, 4.56) ***	-0.0039 (-0.0085, 0.0006)
Class 4 (VL persistent with high levels)	5.78 (4.79, 6.77) ***	-0.0103 (-0.0249, 0.0043)
Class 5 (VL suppressed slowly)	4.99 (4.45, 5.53) ***	-0.0102 (-0.0174, -0.0032) **

Derivation and validation cohorts were combined according to the VL trajectories. 273 patients (out of 1885, 14.5%) had longitudinal data (at least two repeated measurements) of quantitative *HBsAg level for modelling. Specifically, longitudinal data were available for 137, 49, 55, 7, and 25 patients in classes 1 to 5, respectively. In total, 1072 records of HBsAg levels are collected from these 273 patients. The longitudinal trends of HBsAg level over time were assessed by linear mixed effects models with random intercepts and slopes. All linear mixed effects models were adjusted for demographics (including age, sex, and ethnicity). *** indicates p value <0.001, ** indicates p value <0.01. HBV, Hepatitis B Virus; VL, viral load; NA, nucleos/tide analogue; HBsAg, Hepatitis B surface antigen.*

Table S15. The first and most recent measurements of quantitative HBsAg level during follow up for patients with chronic HBV infection on NA therapy in the subset (N=273) of the study cohort

VL trajectory class	Number of patients with qHBsAg level available	Median [IQR] of qHBsAg level for the first measurements	First measurement of qHBsAg level (baseline or during follow up)		Median [IQR] of qHBsAg level for the most recent measurements	The most recent measurement of qHBsAg level	
			**<100 IU/ml**	≥100 IU/ml		**<100 IU/ml**	≥100 IU/ml
Class 1	N=137	3.09 [2.49, 3.60] log10 IU/ml	**17 (12.4)**	120 (87.6)	2.90 [2.32, 3.45] log10 IU/ml	**26 (19.0)**	111 (81.0)
Class 2	N=49	3.58 [2.92, 4.16] log10 IU/ml	**4 (8.2)**	45 (91.8)	3.28 [2.72, 3.77] log10 IU/ml	**7 (14.3)**	42 (85.7)
Class 3	N=55	3.35 [2.86, 3.89] log10 IU/ml	**1 (1.8)**	54 (98.2)	3.32 [2.77, 3.84] log10 IU/ml	**4 (7.3)**	51 (92.7)
Class 4	N=7	4.71 [4.47, 4.92] log10 IU/ml	**0 (0)**	7 (100)	4.58 [4.16, 4.86] log10 IU/ml	**0 (0)**	7 (100)
Class 5	N=25	4.20 [3.55, 4.58] log10 IU/ml	**0 (0)**	25 (100)	3.72 [2.96, 4.21] log10 IU/ml	**0 (0)**	25 (100)

Table S16. Classification performance in validation cohort using a smaller number of VL measurements with restrictions on minimum follow up duration (≥6 months)

	Use all VL measurements available	Use the first two VL measurements	Use the first three VL measurements	Use the first four VL measurements	Use the first five VL measurements
Number of patients	518	260	378	394	359
Follow up duration (months) of VL measurements, median [IQR]	49 [25, 77]	12 [7, 19]	14 [9, 24]	19 [12, 31]	25 [17, 37]
Discrimination	0.9287	0.8323	0.8268	0.8487	0.8667
Entropy	0.8815	0.7307	0.7350	0.7678	0.7922
APPA for each class (class 1: class 2: class 3: class 4: class 5)	0.9370: 0.8643: 0.9224: 0.9751: 0.9403	0.8809: 0.6221: 0.8342: 0.8162: 0.7402	0.8845: 0.6523: 0.8365: 0.7958: 0.7594	0.8916 0.7016 0.8709 0.8729 0.7848	0.8865: 0.7657: 0.8936: 0.8841: 0.8283
Proportions per class (class 1: class 2: class 3: class 4: class 5)	53.86%: 8.69%: 24.9%: 3.67%: 8.88%	61.54%: 8.85%: 10.77%: 7.69%: 11.15%	51.59%: 12.7%: 17.46%: 8.2%: 10.05%	45.43%: 13.45%: 22.84%: 7.87%: 10.41%	43.18%: 13.37%: 24.51%: 10.03%: 8.91%

APPA, average of maximum posterior probability of assignments.

Table S17. Classification performance in validation cohort using a smaller number of VL measurements without restriction on minimum follow up duration

	Use all VL measurements available	Use the first two VL measurements	Use the first three VL measurements	Use the first four VL measurements	Use the first six VL measurements
Number of patients	518	518	469	421	369
Follow up duration (months) of VL measurements, median [IQR]	49 [25, 77]	6 [3, 12]	12 [6, 21]	19 [11, 30]	24 [16, 37]
Discrimination	0.9287	0.7835	0.8160	0.8481	0.8666
Entropy	0.8815	0.6660	0.7160	0.7642	0.7909
APPA for each class	0.9370: 0.8643: 0.9224: 0.9751: 0.9403	0.861: 0.572: 0.719: 0.745: 0.661	0.8765: 0.6382: 0.8111: 0.7850: 0.7446	0.8897: 0.7022: 0.8689: 0.8734: 0.7786	0.8850: 0.7657 0.8933 0.8841 0.8283
Proportions per class	53.86%: 8.69%: 24.9%: 3.67%: 8.88%	55.98%: 7.34%: 19.11%: 7.34%: 10.23%	49.89%: 11.3%: 21.75%: 7.68%: 9.38%	45.61% 12.83% 23.75% 7.6% 10.21%	43.09% 13.01% 25.47% 9.76% 8.67%

APPA, average of maximum posterior probability of assignments.

Table S18. Relationship between VL classes and EASL definition of virological breakthrough

	Virological breakthrough†
Overall cohort (n=1885)	120/1885 (6.4%)
Class 1 (n=1106)	44/1106 (4.0%)
Class 2 (n=383)	11/383 (2.9%)
Class 3 (n=185)	32/185 (17.3%)
Class 4 (n=63)	19/63 (30.2%)
Class 5 (n=148)	14/148 (9.5%)

† *Virological breakthrough: a confirmed increase in HBV DNA level of more than 1 log10 IU/ml compared to the nadir HBV DNA level on-therapy.*
