# Supplementary material for: Distinct virologic trajectories in chronic hepatitis B identify heterogeneity in response to nucleos(t)ide analogue therapy
Source: JHEP Rep. 2024 Oct 9;7(1):101229. doi: 10.1016/j.jhepr.2024.101229 (PMC11664071; doi:10.1016/j.jhepr.2024.101229)
Supplement: Multimedia component 2 [file mmc2.pdf]

JOURNAL
OF HEPATOLOGY

Journal of Hepatology

CTAT methods

Tables for a “Complete, Transparent, Accurate and Timely account” (CTAT) are now mandatory for all revised submissions. The aim is to enhance the reproducibility of methods.

- Only include the parts relevant to your study
- Refer to the CTAT in the main text as ‘Supplementary CTAT Table’
- Do not add subheadings
- Add as many rows as needed to include all information
- Only include one item per row

If the CTAT form is not relevant to your study, please outline the reasons why:

1.1 Antibodies

Name	Citation	Supplier	Cat no.	Clone no.
N/A				

1.2 Cell lines

Name	Citation	Supplier	Cat no.	Passage no.	Authentication test method
N/A					

1.3 Organisms

Name	Citation	Supplier	Strain	Sex	Age	Overall n number
N/A						

1.4 Sequence based reagents

Name	Sequence	Supplier

1.5 Biological samples

Description	Source	Identifier
N/A		

1.6 Deposited data

Name of repository	Identifier	Link
N/A		

1.7 Software

Software name	Manufacturer	Version
R Studio for Window	RStudio, PBC	2022.07.2.576
R	R Foundation for Statistical Computing	Version 4.2.2

1.8 Other (e.g. drugs, proteins, vectors etc.)

NA		

1.9 Please provide the details of the corresponding methods author for the manuscript:

Tingyan Wang
tingyan.wang@ndm.ox.ac.uk

2.0 Please confirm for randomised controlled trials all versions of the clinical protocol are included in the submission. These will be published online as supplementary information.

N/A
